# What are the core recommendations for rheumatoid arthritis care? Systematic review of clinical practice guidelines

**DOI:** 10.1007/s10067-023-06654-0

**Published:** 2023-06-09

**Authors:** Brooke Conley, Samantha Bunzli, Jonathan Bullen, Penny O’Brien, Jennifer Persaud, Tilini Gunatillake, Mandana Nikpour, Rebecca Grainger, Cheryl Barnabe, Ivan Lin

**Affiliations:** 1 Department of Physiotherapy, The University of Melbourne, Melbourne, VIC Australia; 2 School of Health Sciences and Social Work, Griffith University, Brisbane, QLD Australia; 3 Physiotherapy Department, Royal Brisbane and Women’s Hospital, Brisbane, QLD Australia; 4 EnAble Institute, Curtin University, Perth, WA Australia; 5 Department of Surgery, The University of Melbourne, St Vincent’s Hospital Melbourne, Melbourne, VIC Australia; 6 Arthritis and Osteoporosis Western Australia, Perth, WA Australia; 7 Physiotherapy Department, Sir Charles Gairdner Hospital, Nedlands, WA Australia; 8 Departments of Medicine and Rheumatology Melbourne, The University of Melbourne at St. Vincent’s Hospital, Melbourne, VIC Australia; 9 Department of Medicine, University of Otago Wellington, Wellington, New Zealand; 10 Te Whatu Ora Health New Zealand – Capital Coast and Hutt Valley, Wellington, New Zealand; 11 Departments of Medicine and Community Health Sciences, Cumming School of Medicine, University of Calgary, Calgary, AB Canada; 12 Western Australian Centre for Rural Health, The University of Western Australia, Geraldton, WA Australia; 13 Geraldton Regional Aboriginal Medical Service, Geraldton, WA Australia

**Keywords:** Evidence-based care, Rheumatoid arthritis, Practice guidelines, Evidence-based medicine, Systematic review

## Abstract

**Abstract:**

Systematic r eview to evaluate the quality of the clinical practice guidelines (CPG) for rheumatoid arthritis (RA) management and to provide a synthesis of high-quality CPG recommendations, highlighting areas of consistency, and inconsistency. Electronic searches of five databases and four online guideline repositories were performed. RA management CPGs were eligible for inclusion if they were written in English and published between January 2015 and February 2022; focused on adults ≥ 18 years of age; met the criteria of a CPG as defined by the Institute of Medicine; and were rated as high quality on the Appraisal of Guidelines for Research and Evaluation II instrument. RA CPGs were excluded if they required additional payment to access; only addressed recommendations for the system/organization of care and did not include interventional management recommendations; and/or included other arthritic conditions. Of 27 CPGs identified, 13 CPGs met eligibility criteria and were included. Non-pharmacological care should include patient education, patient-centered care, shared decision-making, exercise, orthoses, and a multi-disciplinary approach to care. Pharmacological care should include conventional synthetic disease modifying anti-rheumatic drugs (DMARDs), with methotrexate as the first-line choice. If monotherapy conventional synthetic DMARDs fail to achieve a treatment target, this should be followed by combination therapy conventional synthetic DMARDs (leflunomide, sulfasalazine, hydroxychloroquine), biologic DMARDS and targeted synthetic DMARDS. Management should also include monitoring, pre-treatment investigations and vaccinations, and screening for tuberculosis and hepatitis. Surgical care should be recommended if non-surgical care fails. This synthesis offers clear guidance of evidence-based RA care to healthcare providers.

**Trial registration:**

The protocol for this review was registered with Open Science Framework (https://doi.org/10.17605/OSF.IO/UB3Y7).

**Supplementary Information:**

The online version contains supplementary material available at 10.1007/s10067-023-06654-0.

## Introduction

Rheumatoid arthritis (RA) is a systemic autoimmune disorder affecting 0.1–2.0% of most populations [1]. It is a long-term condition characterized by joint inflammation, with potential for joint damage and extra-articular manifestations [2]. RA can significantly impact physical, mental, and social health and can increase morbidity and mortality [3, 4]. Economic costs, including direct (e.g., drug costs) and indirect costs (e.g., absenteeism and work disability) are estimated to range from US$2,408 to US$83,845 annually [5].

The last 30 years have seen many substantive changes in RA management including expansion in options for pharmacological management, introduction of instruments for clinical monitoring of disease activity and impact, and increased focus on patient-centered care and support for self-management [6, 7]. The pharmacological management options have expanded from conventional synthetic disease-modifying anti-rheumatic drugs (csDMARDs) to biologic DMARDS (bDMARDs) and more recently targeted synthetic DMARDS (tsDMARDS). There are also multiple treatment strategy trials to be considered alongside local medication availability [8]. The evidence underpinning these management approaches are often summarized for clinicians in clinical practice guidelines (CPG).

The aim of CPGs is to support evidence-based clinical decision-making, improve consistency of care and optimize patient outcomes [9]. Robust CPGs comprise a set of management recommendations, created from a systematic review of the literature and consensus by an expert panel [10]. While CPG production has increased in recent decades there are some concerns about quality and implementation into practice [11-13]. Low quality CPGs do not improve care, and conflicting recommendations between CPGs can lead to clinician confusion [11, 14]. To date, systematic reviews have either appraised CPG quality [15], or provided a narrative summary on RA management options [16], or reported on both CPG quality and content, but were only specific for certain management options (e.g., physiotherapy interventions [12], Chinese medicine [17]). To the best of the authors knowledge, currently no systematic review has appraised CPG quality and synthesized recommendations from high-quality CPGs for all management options. By summarizing high quality CPG recommendations, we can offer healthcare providers clear, simple guidance on evidence-based RA care.

The aims of this systematic review were to (1) evaluate the quality of the CPGs for the management of RA and (2) to provide a synthesis of high-quality CPG recommendations, highlighting areas of consistency and inconsistency.

## Materials and methods

This systematic review is reported according to the Preferred Reporting Items for Systematic reviews guidelines and the protocol registered on the Open Science Framework (https://doi.org/10.17605/OSF.IO/UB3Y7) [18, 19]. For full details of methods, see Conley et al. [20]. Briefly, five databases (OvidSP MEDLINE, Cochrane, CINAHL, Embase, and Physiotherapy Evidence Database (PEDro) and four guideline repositories were searched from January 2015 to April 1st, 2023. Online Resource 1 provides the details of the search strategy. Search results were exported into Endnote™ and duplicates removed electronically and manually checked before importing titles/abstracts into Covidence systematic review software (Veritas Health Innovation, Melbourne, Australia. Available at www.covidence.org). Two independent authors (BC and TG or IL) screened the titles and abstracts to identify relevant studies. Then full texts were then screened for eligibility (Table 1). Any discrepancies were resolved by a third reviewer. Deviations from the original protocol included updating the search twice and inclusion of CPGs that addressed one treatment modality (e.g., medication prescribing) which were originally excluded based on their narrow scope.

**Table 1:** Clinical practice guidelines (CPGs) selection criteria

Inclusion criteria • Developed between January 2015 and April 1st, 2023 • For the interventional management of rheumatoid arthritis • For adult populations (aged ≥ 18 years) • Published in the English language or in which a complete English language version is available • Developed using a systematic process that is a guideline based on a systematic review of the literature and developed by an expert, multidisciplinary panel [2] • Represents an original body of work, i.e., not solely an adaptation or systematic review of existing guidelines
Exclusion criteria • Does not include interventional management recommendations • Includes other arthritic conditions • Only addresses recommendations for the system/organisation of care • Unavailable via institutional access, i.e., requires additional payment

The Appraisal of Guidelines for Research and Evaluation (AGREE) II instrument was used to assess CPG quality [21]. This is a valid, reliable tool that is widely used in CPG appraisal, including those for RA management [11, 12, 15, 22, 23]. Pairs of reviewers (from SB, PO, JB, JP, TG, IL, BC) independently rated each CPG against the AGREE II items using a 7-point Likert scale, from 1 (AGREE II criteria not addressed) to 7 (all AGREE II criteria addressed). Individual reviewer domain scores were calculated and expressed as a percentage. We defined acceptable inter-rater agreement as domain scores of 80% or above, consistent with excellent intraclass coefficient values [24, 25]. If the two reviewer’s domain scores varied equal to or greater than 20%, reviewers met to discuss discrepancies and a third reviewer was consulted to resolve any disagreements on the final rating. The AGREE II developers do not provide criteria for CPG quality, rather, they suggest this is at the discretion of the researchers [21]. Consistent with a previous musculoskeletal review, the authors of this study considered the following domains most important when screening high quality RA CPGs: stakeholder involvement (domain 2); rigor of development (domain 3); and editorial independence (domain 6) [13]. Arthritis reviews implementing the AGREE II instrument established an threshold for high quality as equal to or greater than 60% [12, 26]. We decided that CPGs that did not meet this definition were excluded (Online Resource 3).

The first author (BC) independently extracted and recorded CPG data on a bespoke Microsoft Excel spreadsheet, based on a previous musculoskeletal review [13] (Online Resource 2). CPG recommendations were extracted and ranked as either a “should do,” “could do,” “do not do,” or “uncertain” (Online Resource 4). Recommendations were classified into these four categories based on language used in the CPGs (Table 2), consistent with a previous musculoskeletal systematic review of CPGs [13]. Two authors (SB and IL) cross-checked the extracted data and recommendation rankings, any inconsistencies were resolved through discussion among the three authors (BC, SB, IL) while referring to the original CPG.

**Table 2:** Recommendation classification, definition, and examples of terminology for each classification

Recommendation classification	Definition [13, 20]	Examples of terminology from CPGs
“Should do”	“Should do” recommendations were those that the authors determined should be applied in all circumstances unless there is a rationale not to. These were based on strong evidence, for example, multiple high-quality studies reporting clinically relevant positive effects, benefits that outweigh risks or when in the opinion of CPG development group members that the benefits were unequivocal	“Should” [8, 27-34], “strongly recommended”[35]
“Could do”	“Could do” recommendations were those that the authors determined could be applied depending on the circumstances of individual patients. They were usually based on consistent evidence from multiple lesser quality studies or one high quality study and where benefits outweigh harms	“May” [8, 28-30, 34], “could” [27], “can” [28, 30, 31], “consider” [32, 33], “conditionally recommended” [35], suggest offering [36]
“Do not do”	“Do not do” recommendations were those for which the authors determined there was strong evidence of no benefit and/or harms outweighing benefits	“Not recommended”[27, 30, 37], “recommend against” [36]
“Uncertain”	“Uncertain” recommendations were those for which the authors determined there was no recommendation for or against a practice, because of incomplete or inconsistent research findings. Not all CPGs provided uncertain recommendations	“It is not possible to recommend” [27]

**Fig. 1:**
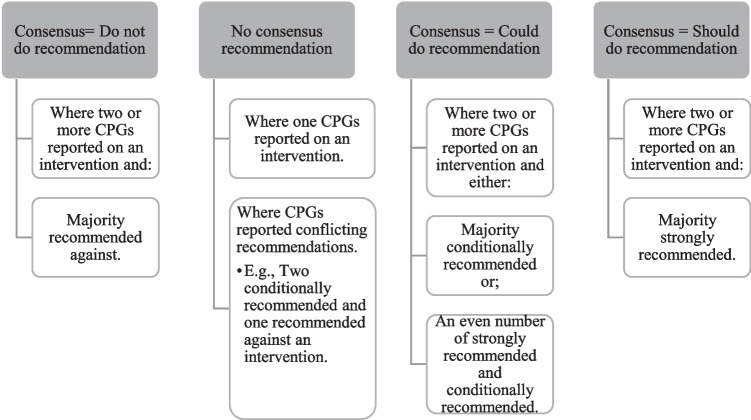
Creating the consensus recommendations

Recommendations were categorized based on the type of intervention (non-pharmacological, pharmacological, and surgical) and then further divided into individual interventions within these categories (e.g., patient education). Narrative summaries were developed for individual interventions and identified which CPGs included a recommendation within that category; and areas of consistency and inconsistency between CPG recommendations (Online Resource 5). The research team developed a consensus process (Fig. 1), to describe the consistency of recommendations between CPGs, providing a global consensus recommendation on the individual interventions.

## Results

**Fig. 2:**
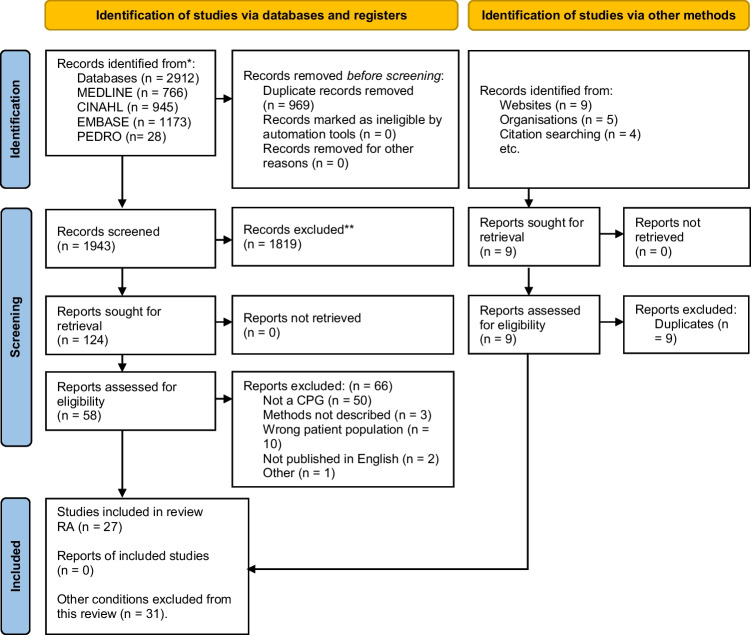
PRISMA 2020 flow diagram for new systematic reviews which included searches of databases, registers, and other sources

Twenty-seven CPGs were identified (Fig. 2). Eleven were excluded as they did not meet criteria of high quality [38-48]. Three CPGs identified earlier in the search period were excluded as two guideline development groups published an updated CPG within our search period [49-51] (Online Resource 3). Thirteen CPGs met the eligibility criteria [4, 8, 27-37]. Six were published in European countries [4, 8, 27, 31, 32, 34], two were published in the USA [33, 35], one in the UK [37], one in Brazil [28], one in Malaysia [29], one in Canada [36] and one internationally (Australia, India, Japan, and New Zealand) [30]. Most CPGs were developed by medical societies (77%) [4, 27-30, 32-36], some by an expert panel (15%) [8, 31] and one was a government report (8%) [37]. Target users were stated as: rheumatologists (*n* = 8) [4, 8, 27, 28, 30, 31, 34, 36]; and other health professionals who manage patients with RA (*n* = 13) [4, 8, 27-37]; patients (*n* = 7) [4, 27, 29, 34-37], their families/carers (*n* = 3) [27, 29, 37]; decision or policy makers (*n* = 3) [4, 29, 36]; those responsible for commissioning care (*n* = 3) [4, 34, 37]; and professional societies (*n* = 1) [29] (Online Resource 2).

Table 3 shows the AGREE II scores for each CPG. Quality was assessed across six domains: scope and purpose (range: 39–97%), stakeholder involvement (range: 42–97%), rigor of development (range: 45–90%), clarity of presentation (range: 56–92%), applicability (range: 19–85%), and editorial independence (range: 58–100%).

**Table 3:** CPG AGREE II domain scores and quality assessment (%) included studies

	Domain 1 Scope and Purpose	Domain 2 Stakeholder involvement	Domain 3 Rigour of development	Domain 4 Clarity of presentation	Domain 5 Applicability	Domain 6 Editorial independence	Overall assessment score	Domain 2,3,6 combined value
Included based on high-quality (13)								
ACR (2021) [35]	92	94	74	83	27	100	78	89
APLAR (2015) [30]	75	72	61	83	52	75	58	70
BSR [28]	78	42	65	83	38	75	67	60
CRA [36]	97	91	76	72	85	79	83	82
EULAR (2023) [34]	72	67	71	86	42	63	75	67
ISR [4]	72	97	76	69	21	92	83	88
MaHTAS [29]	94	69	51	69	50	58	58	60
NICE [37]	92	72	90	92	65	67	83	76
*Peter et al. [33]	64	69	70	67	23	92	64	77
*Santos et al. [32]	83	61	64	56	19	71	59	65
SER (2019) [27]	97	86	63	86	69	58	75	69
*Tenten-Diepenmaat et al. [31]	78	64	45	81	21	71	58	60
TLAR [8]	39	42	56	83	33	96	50	65

^*^First author given where there is no stated organisation; *ACR* American College of Rheumatology, *APLAR* Asia Pacific League of Associations for Rheumatology, *BSR* Brazilian Society of Rheumatology, *CRA* Canadian Rheumatology Association, *EULAR* European League Against Rheumatism, *ISR* Italian Society of Rheumatology, *NICE* National Institute for Health and Care Excellence, *SER* Spanish Society of Rheumatology, *TLAR* Turkish League Against Rheumatism

### Consensus recommendations

**Fig. 3:**
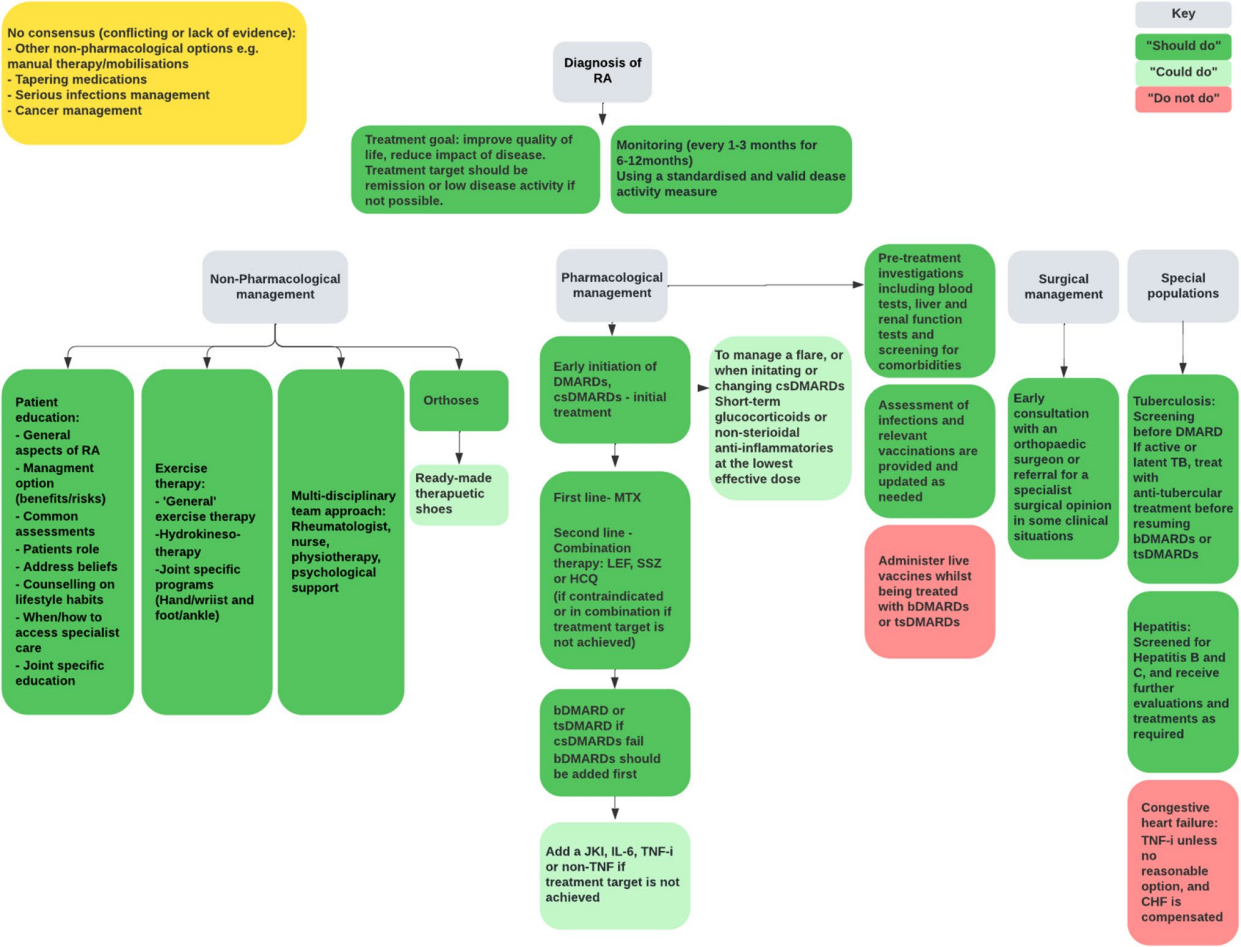
Treatment pathway

Following synthesis, twenty-two common/consistent “should do” recommendations, seven common/consistent “could do” recommendations, two common/consistent “do not do” recommendations and four “no consensus” recommendations were identified (Fig. 3; Online Resource 5).

### Recommendations with “Should do” consensus

The following care elements were strongly recommended by two or more CPGs:

#### Non-pharmacological

Patients and clinicians should adhere to a shared decision-making process and care should be tailored to the patient and their circumstances [8, 28, 30-32, 34, 35, 37] (e.g., considering disease activity [8, 34] and comorbidities [8, 30, 34]). All patients should receive education [27-29, 31-33, 37]. Patient education should include information about the disease and its management options [32, 33, 37] including joint specific care where relevant [29, 31] (e.g., advice on footcare and hygiene). Clinicians should explain the benefits and risks of management options [31, 37]. Clinicians should discuss the importance of healthy lifestyle habits (e.g., exercise, decreasing stress, and fatigue), providing advice on how this can be achieved and maintained [28, 33]. Exercise therapy should be recommended [29, 31-33, 37], with modalities including “general” exercise therapy [31, 32], hydro-kinesiotherapy [32], and joint-specific programs e.g., hand and wrist programs [29, 37] or foot and ankle programs [31]. Foot orthoses [31, 32]/ functional insoles or therapeutic footwear [37] should be recommended for people with RA with abnormal foot function, when adequate over-the-counter shoes are insufficient in reducing foot pain or symptoms. A multi-disciplinary team approach to care should be recommended [8, 28, 29, 31, 32, 37] and should include a rheumatologist [8, 28, 29, 31, 34, 37], nurse [27, 29, 31, 37], physiotherapist [33, 37], and/or psychological support [32, 37] where appropriate.

#### Pharmacological

##### Treatment target and monitoring

Treatment should focus on assisting patients in maximizing their overall quality of life and participation through optimized control of disease activity and maintaining physical function [30, 32]. The treatment goal should be to achieve clinical remission or if that is not possible, low disease activity [8, 28-30, 34, 37]. Validated instruments to measure disease activity were recommended [35], and include disease activity score 28 joints (DAS28) [4, 8, 29, 30, 37], simplified disease activity index (SDAI) [4, 8, 27, 29, 30], clinical disease activity index (CDAI) [4, 8, 27, 29, 30], or other measures such as ACR-EULAR criteria [28-30, 34]. Several CPGs did not describe a preferred instrument or provide a definition of remission/low disease activity [31-33, 35, 36]. Disease activity should be monitored every 1–3 months after diagnosis or if changing treatment strategies until the treatment target is achieved [30, 34, 35]. Once target disease control is reached, patients can be monitored every 3–6 months if disease remains stable, with a review at 6 months [30, 37]. If the treatment target is not achieved by 6 months, therapy should be adjusted [34, 37].

##### Disease-modifying antirheumatic drugs (DMARDs)

Early initiation of DMARDs, “soon after” RA diagnosis [8, 34] is recommended with initial treatment with csDMARDs [4, 8, 27-30, 34, 35, 37]. Methotrexate (MTX) is the preferred csDMARD in all CPGs that reported on pharmacological management [4, 8, 27-30, 34, 35, 37]. If MTX is contraindicated, the patient has intolerance to MTX or does not achieve treatment target using MTX, the csDMARDS, leflunomide (LEF), sulfasalazine (SSZ), or hydroxychloroquine (HCQ) should be recommended [4, 8, 27, 28, 30, 34, 35, 37]. CPGs often failed to report suggested dosages for MTX LEF, SSZ, and HCQ within the recommendations. Two CPGs reported on dosage for MTX, although differed from least 10 mg/week [27] to at least 15 mg/week [35]. bDMARDs or tsDMARDs should be added in conjunction with csDMARDs if treatment target is still not reached [4, 8, 27-30, 34] with bDMARDs being recommended in the first instance [8, 28, 29, 34, 39].

##### Pre-treatment investigations and vaccinations 

Patients should undergo investigations before commencing treatment [29, 30]. These can include screening for comorbidities, pregnancy, chest radiography, blood tests, renal, and liver function tests [29, 30]. All patients should be assessed for infections and vaccinations (particularly live vaccinations) should be provided ideally 4 weeks prior to bDMARD or tsDMARD therapy and updated as needed [4, 28, 30]. Killed or recombinant vaccines can be administered before initiating or during csDMARD, bDMARD, or tsDMARD therapy [4].

##### Special populations

All patients should be screened for tuberculosis (TB) infection before commencing bDMARD or tsDMARD therapy [4, 30]. If a patient has active or latent TB this should be adequately treated before commencing bDMARDs or tsDMARDs [4, 30]. Patients should be screened for hepatitis B virus or hepatitis C virus infections and if positive, receive further evaluation and treatment [4, 30, 35].

#### Surgical

##### Referral for surgical opinion

Surgery should be considered when medical management has not been successful, and the patient meets evidence-based criteria for surgery (Online Resource 5) [31, 37].

### Recommendations with “Could do” consensus

The following recommendations were conditionally recommended by two or more CPGs, or had an equal number of conditionally and strongly recommendations:

Ready-made therapeutic shoes can be considered for patients with RA in certain clinical circumstances, when custom-made shoes are not indicated [31, 37]. If csDMARD therapy fails, janus kinase inhibitor [4, 27, 28, 30, 34], tumor necrosis factor inhibitor (TNF) [4, 8, 28, 34], or non-TNF therapy [4, 27] can be added in conjunction with csDMARDs, while IL-6 inhibitors [8, 27, 34] can be recommended if bDMARDs fail. Non-steroidal anti-inflammatories (NSAIDs) can be added [29, 30, 37] in combination with DMARDs [29]. This includes traditional NSAIDs (+ / − a proton pump inhibitor (PPI) or cox II selective inhibitors [30, 37]. This could be taken orally [8, 37], at the lowest effective dose for the shortest duration [30, 37] to reduce pain and inflammation [8, 29]. Glucocorticoids can be [8, 27-29, 31, 34, 35, 37] considered in response to a patient experiencing a flare/to control active RA [4, 37] or in combination when initiating or changing csDMARDs [4, 8, 27, 29, 30, 34, 37]. Glucocorticoids can include different dose regimens and routes of administration [8, 34], e.g., oral, intramuscular, or intra-articular injections [37]. Injections could be considered for the relief of local symptoms of inflammation [4, 31]. The chosen glucocorticoid should be administered at the lowest dose [4, 28-30, 37] and only used for short-term periods; being tapered when clinically feasible [4, 8, 27, 28, 30, 34] to avoid adverse effects [4, 8]. Definitions of short-term varied among CPGs from < 3 months [29] to < 6 months [4]. Dosage of prednisone recommended varied from ≤ 7.5 [8, 30], ≤ 10 mg once daily [29], or 10–30 mg/day [27].

### Recommendations with “Do not do” consensus

The following were specifically recommended not to be done by two or more CPGs:

TNF inhibitor should not be recommended in persons with a history of congestive heart failure, unless there is no other reasonable option, and the congestive heart failure is compensated [4, 35]. Live vaccines should not be administered while a patient is being treated with bDMARDs or tsDMARDs [4, 30].

### Recommendations with no consensus

CPGs reporting conflicting recommendations for:

Non-pharmacological treatments such as manual therapies (e.g., thermotherapy, massage, dry needling, passive mobilisations [32, 33]) and recommendations for patients with RA and serious infections [27, 30, 35] or cancer [4, 27]. For example, one CPG recommended bDMARDs, with no specific treatment over another [27] while the other CPG recommended csDMARDs for previous low-grade melanoma or non-melanoma skin cancer or lymphoproliferative disorder or standard care for previously treated solid organ malignancy [4]. For full details of conflicting recommendations, see Online Resource 5.

The majority of CPGs suggested pharmacological tapering should or could be considered, occurring after 6 [27, 30, 35, 36] or 12 months [30, 37]; Four CPGs did not advise on a time frame [4, 8, 28, 34]. If a patient is in remission, DMARD treatment could be tapered, in the following order: glucocorticoids, bDMARDs and tsDMARDs and lastly, csDMARDS in certain clinical circumstances [4, 8, 27, 28, 30, 34-37]. However, one CPG recommended against tapering if the patient does not have rapid access to care or will experience difficulty re-establishing access to medications [36].

## Discussion

Clinicians should be aware of and utilize high-quality CPGs to inform decision-making. We determined sixteen of the twenty-seven CPGs were high quality on the AGREE II instrument. CPGs are costly and time-consuming to develop and the significant number of low-quality CPGs is an inefficient use of resources [52]. Local adaption of contemporary high-quality CPGs that includes relevant updates in evidence and a section for area-specific recommendations/considerations such as, availability or cost of treatments may be a more efficient use of resources than development of several original CPGs within a similar time period.

Development of high-quality CPGs are important, although this does not guarantee translation of recommendations into practice [53]. Studies have reported sub-optimal adherence to CPGs, varying from 22 to 100% for rheumatologists with findings being similar across most health disciplines and internationally [54, 55]. The AGREE II instrument measures CPG implementation within the “applicability” domain, which was the lowest scored domain across CPGs (Table 3); consistent with previous systematic reviews for RA management [15, 56-58]. This highlights the need for CPG developers to focus on addressing implementation in future guidelines. Inclusion of economic evaluations, treatment algorithms and monitoring and auditing criteria can facilitate translation of recommendations into practice [21]. A variety of individual, health system and contextual barriers to CPG implementation have been identified by Correa et al. [59], which include insufficient high quality evidence, contradictory CPG, and patient and physician factors [59]. While clinicians may choose to use treatment recommendations developed by local peak bodies, systematic reviews such as ours are important to address concerns about the quality of recommendations, and conflicting recommendations. By applying these recommendations, clinicians in any setting can be confident that they are offering high quality care that is supported by robust evidence.

Recommendations across CPGs were relatively consistent for non-pharmacological, pharmacological, and surgical care. Non-pharmacological interventions should include patient education, patient-centered care, shared decision-making, exercise, orthoses, and a multi-disciplinary approach to care. Pharmacological interventions should include csDMARDs, with MTX as the first-line choice. Followed by csDMARDs as combination therapy (LEF, SSZ and HCQ), bDMARDS, and tsDMARDS to achieve a treatment target. Other aspects of medical management consistently recommended included monitoring, pre-treatment investigations and vaccinations, and screening for tuberculosis and hepatitis. Surgical care should be recommended if non-surgical care fails. TNF inhibitors should not be used in persons with a history of congestive heart failure, unless there is no other reasonable option, and the congestive heart failure is compensated. Live vaccines should be avoided while patients being treated with bDMARDs or tsDMARDs.

Despite the majority of recommendations being consistent across CPGs, they often lacked sufficient detail to guide practice. For example, physiotherapy, psychology, nursing, and rheumatology are professions which provide a range of care options; however, in some CPGs these were broadly classified as interventions. Distinct recommendations for interventions provided by these professions are needed in future CPGs [56]. Similarly, pharmacological recommendations neglected important information needed for implementation into clinical practice such as medication dosage. Dosage for csDMARDs and other medications were often not included with only two CPGs reporting on dosage for MTX, differing from least 10 mg/week to at least 15 mg/week [27, 35]. While dose variation could be attributed to different disease states and consideration of potential side effects, inconsistent dosages, or not including medication dosages are likely barriers to implementation [60]. CPGs often agreed on monitoring frequency [30, 34, 35, 37], and instruments to measure disease activity [4, 8, 27-30, 37, 51] although recommendations on blood monitoring were vague. Medication management is a large component of RA care, and these medications can be associated with adverse effects such as fatigue, nausea, cytopenia, among others [61]. Furthermore, the presence of side effects can prompt changing of treatments. Future CPGs should focus on providing clear, detailed recommendations to improve consistency of care.

No consensus recommendations could be developed due to conflicting recommendations for certain non-pharmacological interventions such as: thermotherapy, recommendations for tapering medications and recommendations for patients with RA and cancer or serious infections. For example, one CPG recommended specific csDMARDs or standard care for patients with RA and certain cancers [4], while the other CPG recommended bDMARDs on a case-by-case basis for patients with RA and cancer [27]. Differences might be attributed to their varied definitions of cancer, either defining it broadly [27] or stating specific cancer types [4]. Guideline development groups interpretation of evidence can influence recommendations [16]. Both CPGs included relevant health professionals in the guideline development group such as rheumatologists and methodological experts, although varied at times for other members, e.g., one CPG included a biostatistician [4] while the other included patients [27]. Another potential reason for the difference could be their methodological process of determining evidence quality, which varied from using either Scottish Intercollegiate Guidelines Network and Osteba critical appraisal tools [27] or the Oxford Levels of Evidence [4].

Our synthesis identified several areas for further development/investigation. Data related to medication tapering is emerging [62]. As such, not all CPGs reported on tapering and those that did differed on timeframes of when to begin tapering. CPGs recommended tapering from 6 [27, 30, 35, 36] or 12 months [30, 37], or did not advise on a time frame [4, 8, 28, 34]. It is important that future CPGs include recommendations on tapering as increasingly people are diagnosed early with RA and treated earlier and the potential issues with long term immunosuppression such as increased likelihood of developing infections [63, 64]. Further research is warranted to explore when tapering should occur and to inform CPGs through quantifying risk of flare with treatment tapering. tsDMARD evidence is an emerging area of research, with JAK-inhibitors have been supported in a recent CPG [34, 65]. Benefits of JAK-inhibitors include their effectiveness and safety, that they can be administered orally and are associated with a lower production cost in comparison to bDMARDs [2]. This highlights the importance of CPGs being updated every 5 years to reflect advances in medicine [10].

## Strengths and potential limitations

Strengths of this systematic review include the use of AGREE II tool as a systematic approach to synthesis [21], and selection of a high-level quality cut-off value, that was based on other reviews in the field [12, 26]. Additionally, we involved a multi-disciplinary team, including rheumatologists (RG, MN, CB), physiotherapists (IL, SB, JP, BC), and social scientists (TG, PO, JB).

The AGREE II instrument examines CPG methodology, not necessarily content and scores can be influenced by authors’ reporting rather than methodological quality [20]. Our search strategy may have failed to identify all relevant CPGs in relation to RA care as non-English language CPGs and CPGs that addressed assessment and/or diagnosis of RA without management or treatment recommendations were excluded. To reduce the likelihood of CPGs being missed, a medical reference librarian assisted in the development of the search strategy and all authors checked the list of full-text CPGs to determine if any were missing to the best of their knowledge. Authors were required to interpret the language used in CPG recommendations, to provide grading of interventions (e.g., “should do” and “could do”). To improve confidence in our interpretations, consensus statements were developed by three authors (BC, SB, and IL) and reviewed by the expert clinicians (MN, RG, CB). The process of interpretation is clearly reported in the methods, and in previous reviews [20].

## Conclusion

Sixteen of the twenty-seven CPGs were identified as high quality on the AGREE II instrument.

Thirteen CPGs met the eligibility criteria, and their recommendations were synthesized. Non-pharmacological care should include patient education, patient-centered care, shared decision making, exercise, orthoses, and a multi-disciplinary approach to care. Pharmacological care should include csDMARDs, with MTX as the first-line choice. If monotherapy csDMARDs fail to achieve a treatment target, this should be followed by combination therapy csDMARDs (LEF, SSZ, HCQ), bDMARDS, and tsDMARDS. Management should also include monitoring, pre-treatment investigations and vaccinations, and screening for tuberculosis and hepatitis. Surgical care should be recommended if non-surgical care fails. This synthesis can provide clear, simple guidance of evidence-based RA care to healthcare providers.

## Supplementary Information

Below is the link to the electronic supplementary material.

Supplementary file1 (DOCX 15 KB)

Supplementary file2 (DOCX 22 KB)

Supplementary file3 (DOCX 21 KB)

Supplementary file4 (DOCX 60 KB)

Supplementary file5 (DOCX 187 KB)

Supplementary file6 (DOCX 36 KB)

## Abbreviations

AGREE: Appraisal of guidelines for research and evaluation
bDMARDs: Biologic disease-modifying anti-rheumatic drug
CHF: Congestive heart failure
CPG: Clinical practice guideline
csDMARDs: Conventional synthetic disease-modifying anti-rheumatic drug
DMARD: Disease-modifying anti-rheumatic drug
HCQ: Hydroxychloroquine
LEF: Leflunomide
MTX: Methotrexate
NSAIDs: Non-steroidal anti-inflammatory drugs
RA: Rheumatoid arthritis
SSZ: Sulfasalazine
TB: Tuberculosis
TNF: Tumor necrosis factor inhibitor
tsDMARDs: Targeted synthetic disease-modifying antirheumatic drugs
