# Supplementary material for: What are the core recommendations for rheumatoid arthritis care? Systematic review of clinical practice guidelines
Source: Clin Rheumatol. 2023 Jun 9;42(9):2267–78. doi: 10.1007/s10067-023-06654-0 (PMC10412487; doi:10.1007/s10067-023-06654-0)
Supplement: Supplementary file 1 — Supplementary file1 (DOCX 15 KB) [file 10067_2023_6654_MOESM1_ESM.docx]

**Online Resource 1. Search Strategy**

Database search strategies undertaken 11/2/2021, updated on 14/2/2022 and further updated 1/4/23.

**Medline**

1. guideline*.ti,pt,kw.
2. Practice Guideline/ or Guideline/
3. 1 or 2
4. (arthriti* or osteoarthriti* or lupus or gout).mp.
5. exp arthritis/ or exp Lupus Erythematosus, Systemic/ or Gout/
6. 4 or 5
7. 3 and 6
8. limit 7 to (english language and yr="2015 - 2020")

**CINAHL**

1. MH Practice Guidelines OR TI guideline* OR PT Practice Guidelines
2. MH arthritis+
3. MH Lupus Erythematosus, Systemic+
4. MH Gout
5. (arthriti* or osteoarhriti* or lupus or gout)
6. S2 OR S3 OR S4 OR S5
7. S1 AND S6
8. S1 AND S6 limit to 2015-2020 and english

**EMBASE**

1. Guideline*.ti,kw.
2. *practice guideline/
3. 1 or 2
4. (arthriti* or osteoarthriti* or lupus or gout).ti,ab,kw.
5. exp *arthritis/
6. exp *systemic lupus erythematosus/
7. *gout/
8. 4 or 5 or 6 or 7
9. 3 and 8
10. limit 9 to conference abstracts
11. 9 not 10
12. limit 11 to (english language and yr="2015 - 2020")

**PEDRO**

1. Abstract and title: Osteoarthritis
2. Abstract and title: Rheumatoid arthritis
3. Abstract and title: Lupus / SLE
4. Gout

| Method | Practice guideline |
| --- | --- |
| When searching | Match all terms with and |
| Published since | 2015 |
