# Supplementary material for: What are the core recommendations for rheumatoid arthritis care? Systematic review of clinical practice guidelines
Source: Clin Rheumatol. 2023 Jun 9;42(9):2267–78. doi: 10.1007/s10067-023-06654-0 (PMC10412487; doi:10.1007/s10067-023-06654-0)
Supplement: Supplementary file 2 — Supplementary file2 (DOCX 22 KB) [file 10067_2023_6654_MOESM2_ESM.docx]

**Online resource 2. Description of clinical practice guidelines**

| **Title** | **Year** | **Type of developer** | **Country/Region of Origin** | **Format** | **Informed by CPG quality tool** | **Accompanying documents accessed** | **Guideline topic** | **Target users** |
| --- | --- | --- | --- | --- | --- | --- | --- | --- |
| ACR [48] | 2021 | Medical Society | USA | Journal article | Yes | Appendix 1-6 | Pharmacological treatment of patients with RA | Health care providers and patients with RA. |
| APLAR [45] | 2015 | Medical Society | International - Australia, Indian, Japan and New Zealand | Journal Article | Yes | Nil | RA management practices in the AP region. | Rheumatologists and all practitioners who manage RA. |
| BSR [42] | 2018 | Medical Society | Brazil | Journal Article | No | Additional file 1 | Drug treatment of adults with RA in Brazil. | Brazilian rheumatologists, physicians. |
| CRA [51] | 2022 | Medical Society | Canada | Journal article | Yes | Supplementary Material | Pharmacological management of  RA with disease-modifying anti-rheumatic drugs for  adult patients. This includes patients whose RA began in childhood or adolescence as juvenile idiopathic arthritis and has persisted into adulthood. | Rheumatologists or other primary prescribers of RA medications, patients, in community and academic practice settings.  May also be of interest to other provincial and federal RA stakeholders and decision makers. |
| EULAR [50] | 2023 | Medical Society | Europe | Journal article | Yes | N/A | Management with synthetic and biological  disease-modifying  antirheumatic drugs. | Rheumatologists, health professionals, patients, regulators, payers. |
| ISR [4] | 2019 | Medical Society | Europe - Italy | Journal Article | Yes | Nil | Management and safety of adult patients with RA in Italy. | Attending physicians (general practitioners, rheumatologists) and health professionals who manage patients with RA in primary care, and hospital and community practice settings. Patients, policy makers and those responsible for commissioning care for patients with RA in the Italian National Health Service (NHS). |
| Malaysia  Health Technology Assessment Section  [43] | 2019 | Medical society | Malaysia | Report | Yes | N/a | Diagnosis, investigations, treatment (non-pharmacological and pharmacological), special considerations and referral/follow-up for patients with RA. | Those who are involved in primary, secondary or tertiary care  management of RA: Doctors, allied health professionals, trainees and medical students, policy makers, patients and their advocates and professional societies. |
| NICE [44] | 2018 | Government | UK | Report | Yes | Appendices  Methods document  Online development materials (e.g. implementation support, resource impact, summaries etc) | Diagnosing and managing RA in adults (people who are aged 18 or older). | Healthcare professionals, commissioners and providers, people with RA and their families and carers. |
| Peter et al. [49] | 2021 | Medical Society | USA | Journal article | Yes | Appendix document  Supplementary figure | Physical therapy management for patients with RA | Physical therapists. |
| Santos et al. [47] | 2021 | Medical  Society | Europe - Portugal | Journal article | Yes | Umbrella review | Management of patients with RA | Health professionals. |
| SER [41] | 2019 | Medical Society | Europe - Spain | Report | Yes | Nil | Management of adults with RA | Rheumatologists and other health professionals who may be involved in the care of patients with RA working in primary and specialist care, namely, those from the specialities of cardiology, pulmonology, traumatology, rehabilitation, family medicine, and nursing, as well as other specialists involved in the care for these patients. Patients and family members seen by these health professionals. |
| Tenten-Diepenmaat et al. [46] | 2018 | Expert panel | Europe – The Netherlands | Journal article | Yes | Additional files 1-3  Research questions  Diagnosis framework  Treatment framework | Multidisciplinary  recommendations and frameworks for the diagnosis  and treatment of foot problems in people with RA. | Health professionals who provide footcare for patients with RA e.g. rheumatologists, rehabilitation  physicians, orthopaedic surgeons,  specialised nurses, podiatrists, orthopaedic shoe technicians and pedicurists. |
| TLAR [8] | 2018 | Expert Panel | Europe - Turkey | Journal Article | No | Nil | Management of RA in Turkey. | Turkish rheumatologists and physical medicine and rehabilitation specialists. |

*First author given where there is no stated organisation; ACR – American College of Rheumatology; APLAR – Asia Pacific League of Associations for Rheumatology; BSR – Brazilian Society of Rheumatology; CRA – Canadian Rheumatology Association; EULAR - European League Against Rheumatism; ISR – Italian Society of Rheumatology; NICE - National Institute for Health and Care Excellence; RA – Rheumatoid arthritis; SER – Spanish Society of Rheumatology; TLAR – Turkish League Against Rheumatism; USA – United States of America; UK – United Kingdom.

Adults are defined as: ≥18 years of age.
