# Supplementary material for: What are the core recommendations for rheumatoid arthritis care? Systematic review of clinical practice guidelines
Source: Clin Rheumatol. 2023 Jun 9;42(9):2267–78. doi: 10.1007/s10067-023-06654-0 (PMC10412487; doi:10.1007/s10067-023-06654-0)
Supplement: Supplementary file 3 — Supplementary file3 (DOCX 21 KB) [file 10067_2023_6654_MOESM3_ESM.docx]

**Online Resource 3. Clinical practice guideline AGREE II domain scores and quality assessment (%) excluded studies**

|  | **Domain 1**  **Scope and Purpose** | **Domain 2**  **Stakeholder involvement** | **Domain 3**  **Rigour of development** | **Domain 4**  **Clarity of presentation** | **Domain 5**  **Applicability** | **Domain 6**  **Editorial independence** | **Overall assessment score** | **Domain 2,3,6 combined value** |
| --- | --- | --- | --- | --- | --- | --- | --- | --- |
| Removed based on newer versions have been published and included (3) | | | | | | | | |
| ACR (2016) [38] | 81 | 83 | 81 | 83 | 52 | 58 | 75 | 74 |
| EULAR (2017) [39] | 78 | 56 | 73 | 86 | 19 | 67 | 75 | 65 |
| EULAR (2019) [40] | 75 | 69 | 81 | 92 | 27 | 58 | 67 | 70 |
| Removed based on low-quality (11) | | | | | | | | |
| APLAR (2019) [28] | 56 | 36 | 51 | 72 | 8 | 21 | 33 | 36 |
| BSR [34] | 64 | 36 | 32 | 67 | 15 | 17 | 25 | 28 |
| FSR [29] | 42 | 56 | 40 | 75 | 48 | 13 | 33 | 36 |
| *García-Vicuna R, et al. [32] | 67 | 31 | 52 | 67 | 8 | 38 | 42 | 40 |
| JCR [37] | 33 | 19 | 9 | 64 | 17 | 58 | 33 | 29 |
| KAR [30] | 69 | 64 | 46 | 86 | 40 | 50 | 42 | 53 |
| *Lin et al. [36] | 33 | 31 | 51 | 22 | 2 | 25 | 25 | 36 |
| MCR [31] | 50 | 22 | 42 | 78 | 46 | 67 | 42 | 44 |
| SER (2015) [33] | 53 | 42 | 33 | 78 | 33 | 17 | 42 | 31 |
| TRA (2017) [27] | 72 | 44 | 57 | 72 | 23 | 46 | 50 | 49 |
| TRA (2017) bDMARDs/tsDMARDs [35] | 50 | 31 | 35 | 69 | 17 | 29 | 42 | 32 |

*First author given where there is no stated organisation; ACR – American College of Rheumatology; APLAR – Asia Pacific League of Associations for Rheumatology; bDMARDs – biologic disease-modifying anti-rheumatic drugs; BSR – Brazilian Society of Rheumatology; EULAR – European League Against Rheumatism; JCR – Japan College of Rheumatology; ISR – Italian Society of Rheumatology; MaHTAS – Malaysia Health Technology Assessment Section; NICE - National Institute for Health and Care Excellence; SER – Spanish Society of Rheumatology; TLAR – Turkish League Against Rheumatism; TRA - Thai Rheumatism Association; tsDMARDs – targeted synthetic disease-modifying anti-rheumatic drugs; FSR – French Society for rheumatology; KAR - Kuwait association of rheumatology; MCR - Mexican College of Rheumatology; SER – Spanish Society of Rheumatology.
