# Supplementary material for: What are the core recommendations for rheumatoid arthritis care? Systematic review of clinical practice guidelines
Source: Clin Rheumatol. 2023 Jun 9;42(9):2267–78. doi: 10.1007/s10067-023-06654-0 (PMC10412487; doi:10.1007/s10067-023-06654-0)
Supplement: Supplementary file 4 — Supplementary file4 (DOCX 60 KB) [file 10067_2023_6654_MOESM4_ESM.docx]

**Online Resource 4. Classification of Rheumatoid Arthritis Recommendations**

**Table 1. Non-pharmacological recommendations**

|  | ACR (2021) [48] | APLAR [45] | BSR  [42] | CRA  [51] | EULAR  (2023) [50] | ISR  [4] | MaHTAS [43] | NICE [44] | Peter et al. [49] | Santos et al. [47] | SER  [41] | Tenten-Diepenmaat et al. [46] | TLAR  [8] |
| --- | --- | --- | --- | --- | --- | --- | --- | --- | --- | --- | --- | --- | --- |
| Non-Pharmacological interventions |  |  |  |  |  |  |  |  |  | ✓✓ |  |  |  |
| Patient education |  |  | ✓✓ |  |  |  | ✓✓ | ✓✓ | ✓ | ✓✓ | ✓✓ | ✓✓ |  |
| Patient centered care/ Shared decision making | ✓✓ | ✓✓ | ✓✓ |  | ✓✓ |  |  | ✓✓ |  | ✓✓ |  | ✓✓ | ✓✓ |
| Exercise |  |  |  |  |  |  | ✓✓ | ✓ | ✓✓ | ✓✓ |  | ✓✓ (General)  ✓ (Foot & Ankle) |  |
| Diet and complementary therapies |  |  |  |  |  |  |  | ✓ Mediterranean diet  O Replace evidence-based therapies  O Impact clinicians’ attitudes |  |  |  |  |  |
| Other non-pharmacological management strategies (e.g. manual therapy, psychological and social interventions) |  |  |  |  |  |  |  |  | O  ✓ Short term mobilisation  O Cervical problems | ✓ |  |  |  |
| Over-the counter shoes |  |  |  |  |  |  |  |  |  |  |  | ✓✓ Sufficient room toe box and stiff sole  ✓ Additional features |  |
| Ready-made therapeutic shoes |  |  |  |  |  |  |  | ✓✓ |  |  |  | ✓ |  |
| Custom-made therapeutic shoes |  |  |  |  |  |  |  |  |  |  |  | ✓✓ Worn all day after a habituation period  ✓ Shoes |  |
| Foot orthoses |  |  |  |  |  |  |  | ✓✓ |  | ✓✓ |  | ✓✓ |  |
| Rigid foot orthoses |  |  |  |  |  |  |  |  |  |  |  | ✓✓ |  |
| Total contact foot orthoses |  |  |  |  |  |  |  |  |  |  |  | ✓✓ |  |
| Silicone toe orthosis |  |  |  |  |  |  |  |  |  |  |  | ✓ |  |
| Toenail brace |  |  |  |  |  |  |  |  |  |  |  | ✓ |  |
| Multi-disciplinary team |  |  | ✓✓ |  |  |  | ✓ | ✓✓ |  | ✓✓ |  | ✓✓ | ✓✓ |
| Nursing |  |  |  |  |  |  | ✓✓ | ✓✓ |  |  | ✓✓ | ✓✓ |  |
| Physiotherapy |  |  |  |  |  |  |  | ✓✓ | ✓✓ |  |  |  |  |
| Occupational therapy |  |  |  |  |  |  |  | ✓✓ |  |  |  |  |  |
| Podiatry |  |  |  |  |  |  |  | ✓✓ |  |  |  |  |  |
| Psychological |  |  |  |  |  |  |  | ✓✓ |  | ✓✓ |  |  |  |
| Rheumatologist |  |  | ✓✓ |  | ✓✓ |  | ✓✓ | ✓✓ |  |  |  | ✓✓ | ✓✓ |
| Supervised program |  |  |  |  |  |  |  |  |  |  | ✓ |  |  |

✓✓Should do; ✓ Could do; O Do not do.

ACR – American College of Rheumatology; APLAR – Asia Pacific League of Associations for Rheumatology; BSR – Brazilian Society of Rheumatology; CRA – Canadian Rheumatology Association; EULAR – The European League Against Rheumatism; Example; ISR – Italian Society of Rheumatology; MaHTAS – Malaysia Health Technology Assessment Section; NICE – National Institute for Health and Care Excellence; SER – Spanish Rheumatology Society; TLAR – Turkish League Against Rheumatism.

**Table 2. Pharmacological recommendations interventions**

|  | ACR (2021) [1] | APLAR [2] | BSR [3] | CRA [4] | EULAR (2023) [5] | ISR [6] | MaHTAS [7] | NICE [8] | Peter et al. [9] | Santos et al. [10] | SER [11] | Tenten-Diepenmaat et al. [12] | TLAR [13] |  |
| --- | --- | --- | --- | --- | --- | --- | --- | --- | --- | --- | --- | --- | --- | --- |
| Pharmacological interventions |  |  |  |  |  |  |  |  |  | ✓✓ |  |  |  |  |
| Early initiation of DMARDs |  |  |  |  | ✓✓ |  |  |  |  |  |  |  | ✓✓ |  |
| Initial treatment with conventional synthetic disease modifying antirheumatic drugs (csDMARDs) | | | | | | | | | | | | | |  |
| csDMARDS (first-line) | ✓ Initiation of a csDMARD without short-term GCs(<3 months) OR  ✓✓ without longer-term (≥3 months) GCs | ✓✓ Monotherapy | ✓✓ Monotherapy |  | ✓✓ Monotherapy | ✓✓ | ✓✓ Monotherapy | ✓✓ Monotherapy |  |  | ✓✓ one or several (with GCs) |  | ✓✓ Mono-therapy |  |
| Selection of csDMARDs | | | | | | | | | | | | | |  |
| Methotrexate (MTX) | ✓✓ and ✓ MTX vs other pharmacological options ✓ MTX oral ✓ Switching to subcutaneous ✓ MTX 15 mg per week ✓ A split dose of oral methotrexate | ✓✓ First line | ✓✓ First line |  | ✓✓ First line | ✓✓ First line | ✓✓ First line | ✓✓ First line |  |  | ✓✓ First line |  | ✓✓ First line |  |
| Leflunomide (LEF), sulfasalazine (SSZ) and hydroxychloroquine (HCQ) | ✓ HCQ ✓ SSZ | ✓ LEF, SSZ or HCQ | ✓✓ MTX with LEF or HCQ and SSZ OR switching MTX for another csDMARD (LLF or SSZ) alone |  | ✓✓ LEF and SSZ | ✓✓ LEF and SSZ |  | ✓✓ LEF and SSZ  ✓ HCQ alternative if mild or palindromic disease |  |  | ✓✓ LEF + Biologic |  | ✓✓ LEF SSZ |  |
| csDMARDs (second-line) |  | ✓✓ Combination therapy MTX and other csDMARD or bDMARDs ✓✓ Triple therapy with cDMARDs | ✓ Combination therapy |  | ✓✓ Combination therapy | ✓✓ other csDMARDs |  | ✓✓ Combination therapy |  |  | ✓ Combination csDMARDs or bDMARD  O Triple therapy initially  ✓ Triple therapy if GCs are contraindicated |  | ✓✓ Combination therapy |  |
| Treatment if csDMARD fails | | | | | | | | | | | | | |  |
| Biologic disease-modifying anti-rheumatic drug (bDMARD)  Targeted synthetic disease-modifying antirheumatic drug (tsDMARD) | ✓ Switch to bDMARD or tsDMARD different class ✓ addition of these over triple therapy | ✓ bDMARD (inadequate response or intolerance csDMARD) ✓ Early consideration for active disease with poor prognostic factors  ✓✓ bDMARDs + MTX ✓✓ switch bDMARD at 6 months if failed to achieve treatment target | ✓ bDMARD or tsDMARD + csDMARD (preferably bDMARD +MTX) ✓ Failure of an initial treatment scheme with bDMARD, swap to another bDMARD  ✓✓ consider safety and cost of each bDMARD |  | ✓✓ bDMARD ✓✓ bDMARD or tsDMARD + csDMARD ✓✓ bDMARD or tsDMARD failure, swap to another bDMARD or tsDMARD | ✓✓ bDMARD or tsDMARD  ✓✓ bDMARD or tsDMARD failure, swap to another bDMARD or tsDMARD | ✓✓ bDMARD and tsDMARD  ✓ bDMARD effective alternative to csDMARD |  |  |  | ✓✓ bDMARD or tsDMARD ✓✓ bDMARD + LEF  ? Specific biological agent  ✓ biologic has failed bDMARD or tsDMARD |  | ✓✓ bDMARD or tsDMARD + csDMARD  ✓✓ bDMARD first line ✓✓ if failure, swap to another bDMARD or tsDMARD |  |
| Janus kinase inhibitor |  | ✓ Tofacitinib | ✓ Tofacitinib |  | ✓ | ✓ |  |  |  |  | ✓ |  |  |  |
| TNF-inhibitor |  |  | ✓ |  | ✓ | ✓ |  |  |  |  |  |  | ✓ |  |
| non-TNF biologic |  |  |  |  |  | ✓ |  |  |  |  | ✓ |  |  |  |
| Interleukin (IL)-6 inhibitors |  |  |  |  | ✓ IL-6 inhibitors or tsDMARDs over other bDMARDs |  |  |  |  |  | ✓✓ |  | ✓ IL-6 inhibitors or tsDMARDs over other bDMARDs |  |
| Anakinra |  |  |  |  |  |  |  | O  ✓ Controlled long term clinical study |  |  |  |  |  |  |
| Treatment Targets | | | | | | | | | | | | | |  |
| Treatment goal | ✓✓ Treat-to-target approach who have not been previously treated with bDMARDs or tsDMARDs  ✓ Treat-to-target approach who have had an inadequate response to bDMARDs or tsDMARDs ✓ Low disease activity goal | ✓✓ Remission or low disease activity  ✓✓ Quality of life and maintaining physical functioning | ✓✓ Remission or low disease activity |  | ✓✓ Remission or low disease activity |  | ✓✓ Treat-to-target strategy  ✓✓ Remission or low disease activity within 6 months | ✓✓ Treat-to-target strategy ✓✓ Remission or low disease activity  ✓ Target remission for people with presence of anti-CCP antibodies or erosions on X-ray at baseline assessment |  | ✓✓ Maximising their overall quality and enjoyment of life, through optimized control of the impact of disease |  |  | ✓✓ Remission or low disease activity |  |
| Monitoring | | | | | | | | | | | | | |  |
| Monitoring | ✓✓ Re-evaluate treatment decisions within 3 months based on efficacy and tolerability of the DMARD(s) chosen. | ✓✓ 1-3 months (recently diagnosis or active disease)  ✓ 3-6 months (remission or low disease activity)  ✓✓ A suitable and practical standardized measure of disease activity ✓✓ Safety monitoring on bDMARD therapy |  |  | ✓✓ 1-3 months |  |  | ✓✓ Ongoing medication monitoring  ✓ Review appointment at 6 months after achieving treatment target  ✓✓ Annual review  O Ultrasound routinely |  | ✓✓ Validated instrument for monitoring |  |  |  |  |
| Tapering | | | | | | | | | | | | | |  |
| Tapering | ✓ Continuing DMARDs at current dose rather than tapering ✓ Dose reduction>gradual discontinuation ✓ Gradual discontinuation> abrupt discontinuation ✓ Gradual discontinuation of SSZ >HCQ (if triple therapy) ✓ Gradual discontinuation of MTX > bDMARD or tsDMARD | ✓✓ In patients who have achieved remission, a reduction in treatment should be considered  ✓ 6 months remission - corticosteroids and NSAIDs tapered ✓ 6 to 12 months after discontinuation of NSAIDs, corticosteroids and bDMARDs, gradual reduction in cDMARDs | ✓ Taper bDMARD | ✓ In patients who have sustained low disease activity or remission for 6 months Tapering bsDMARDs and tsDMARDs  O If rapid access to care or re-establishing access to medications is challenging | ✓ Taper GCs Then dose reduction of DMARDs (bDMARDs/tsDMARDs* and/or csDMARDs) | ✓✓ Sustained remission reduce treatment  ✓ bDMARDs or tsDMARDs tapering then ✓ cDMARD tapering |  | ✓ Reducing drug doses or stopping drugs in a step-down strategy at 12 months w/o glucocorticoids |  |  | ✓✓ 6 months remission progressively taper biologic dose |  | ✓ Tapering GC’s (if using csDMARDs) ✓ Tapering bDMARDs ✓ Tapering cDMARDs if persistent remission |  |
| Adjunctive therapy | | | | | | | | | | | | | |  |
| Non-steroidal anti-inflammatories (NSAIDs) |  | ✓✓ Lowest dose, short-term |  |  |  |  | ✓ | ✓ |  |  |  |  |  |  |
|  |  |  |  |  |  |  |  |  |  |  |  |  |  |  |
|  |  |  |  |  |  |  |  |  |  |  |  |  |  |  |
| Glucocorticoids (GCs) | ✓ Addition of or switching to DMARD > continuation of GCs or over the use of intraarticular GCs alone | ✓✓ Prednisolone ✓✓ Lowest possible dose and tapered as rapidly as clinically feasible  ✓ Oral corticosteroids Active RA in combination with cDMARDs  O Oral corticosteroid monotherapy | ✓✓ Short-term ✓✓ Consider risk-benefit ratio |  | ✓✓ Short-term | ✓ Short-term  ✓✓ Intra-articular glucocorticoid injections | ✓ Short-term | ✓ Short-term ✓ Long-term if complications have been discussed and other treatment options have been offered |  |  | ✓✓ Prednisolone (+ one or several csDMARDs) | ✓ | ✓✓ Short-term use if csDMARD initiation or change |  |
| Pre-treatment investigations | | | | | | | | | | | | | |  |
| Pre-treatment investigations |  | ✓✓ |  |  |  |  | ✓✓ |  |  |  |  |  |  |  |
| Vaccinations |  | ✓✓ All vaccines ideally, especially live vaccines 4 weeks prior to bDMARD  O Live vaccines whilst being treated with bDMARD | ✓✓ Update of record |  |  | ✓✓ All vaccines ideally 4 weeks prior to starting tsDMARD or bDMARD therapy  ✓✓ Killed and recombinant vaccines before initating or during therapy with csDMARDs, tsDMARDs, bDMARDs  O Live vaccines whilst being treated with bDMARD or tsDMARD |  |  |  |  |  |  |  |  |

✓✓Should do; ✓ Could do; ? Uncertain; O Do not do; > rather than.

ACR – American College of Rheumatology; Anti-CCP – Anti-cyclic citrinullated peptide; APLAR – Asia Pacific League of Associations for Rheumatology; BDMARDs – biologic disease-modifying anti-rheumatic drug; BSR – Brazilian Society of Rheumatology; csDMARDs – conventional synthetic disease-modifying anti-rheumatic drug; DMARD – disease-modifying anti-rheumatic drug; EULAR – The European League Against Rheumatism; GCs – glucocorticoids; HCQ – hydroxychloroquine; IL – Interleukin (IL); ISR – Italian Society of Rheumatology; LEF – leflunomide;MaHTAS – Malaysia Health Technology Assessment Section; MTX – methotrexate; NICE – National Institute for Health and Care Excellence; NSAIDs – non-steroidal anti-inflammatories; SER – Spanish Rheumatology Society; SSZ – sulfasalazine; TLAR – Turkish League Against Rheumatism; tsDMARDs – targeted synthetic disease-modifying antirheumatic drugs.

**Table 3. Special populations recommendations**

|  | ACR (2021) [48] | APLAR [45] | BSR  [42] | CRA  [51] | EULAR  (2023) [50] | ISR  [4] | MaHTAS [43] | NICE [44] | Peter et al. [49] | Santos et al. [47] | SER  [41] | Tenten-Diepenmaat et al. [46] | TLAR  [8] |
| --- | --- | --- | --- | --- | --- | --- | --- | --- | --- | --- | --- | --- | --- |
| Interstitial lung disease |  |  |  |  |  |  |  |  |  |  | ✓✓Abatacept  ✓ Rituximab  ? Interstitial pneumonia medications |  |  |
| Infections | ✓ Addition of csDMARDs  ✓ Addition of/switching to DMARDs | O bDMARD |  |  |  |  |  |  |  |  | ✓✓ Abatacept or Etanercept |  |  |
| Foot conditions |  |  |  |  |  |  |  |  |  |  |  | ✓✓ Fungal nail or mycosis - treatment prevent ulcers and secondary bacterial infections ✓✓ Hyperkeratotic lesions - Normalisation of pressure and shearing forces ✓✓ Excessive hyperkeratotic lesions treated ✓✓ Pre-ulcer or infection is detected, consult the treating physician  ✓ Applying a provisional therapy (e.g., felt padding) in wound care |  |
| Cancer |  |  |  |  |  | ✓ csDMARDs rather than tsDMARDs or bDMARDs  Previously treated Lymphoproliferative disorders ✓ Rituximab  ✓ Combination of csDMARDs or Abatacept or Tocilizumab  ✓ Previously treated solid organ malignancy, same response as those without this condition |  |  |  |  | ✓✓ Biological therapy on a case-by-case basis  ? No evidence for a specific biologic |  |  |
| Tuberculosis |  | ✓✓ Screening ✓✓ Latent TB - prophylactic anti-TB therapy  ✓✓ Active TB - adequate treatment before bDMARD |  |  |  | ✓✓ Screening ✓✓ If positive TST or IGRA - further investigations  ✓ If negative, no further workup  ✓✓ Annual testing for at risk areas ✓✓ Active or latent TB, appropriate anti-tubercular treatment  ✓ Referral to specialist ✓ tsDMARDs or bDMARDs following treatment and 1 month of latent TB |  |  |  |  |  |  |  |
| Hepatitis | ✓✓ Prophylactic antiviral therapy vs frequent monitoring  ✓ Frequent monitoring alone of viral load and liver enzymes | ✓✓ Screened for TB, and HBV and HCV infections before initiating bDMARD therapy  O Avoid bDMARDs if pt active or untreated chronic HBV infection and active HCV infection |  |  |  | ✓✓ Screened before bDMARD and tDMARD ✓✓ AntiHBcAb positive or HBsAg negative should undergo further evaluations ✓✓ HBsAg positive should undergo further evaluations ✓✓ Active HBV treated entecavir or tenofovir  ✓✓ Acute HB receive antiviral  ✓✓ Inactive HBV carriers prophylaxis 4 weeks before immunosuppressive  ✓✓ Patients stopping prophylaxis should be closely monitored |  |  |  |  |  |  |  |
| Pregnancy and lactation |  | ✓✓ only consider bDMARDs after assessing benefits/risks |  |  |  |  |  |  |  |  |  |  |  |
| Nontuberculous mycobacterial lung disease | ✓ Lowest possible dose of GCs (discontinuation if possible)  ✓ Addition of csDMARDs  ✓ Abatacept |  |  |  |  |  |  |  |  |  |  |  |  |
| Pulmonary disease | ✓ MTX |  |  |  |  |  |  |  |  |  |  |  |  |
| Congestive heart failure (CHF) | ✓ Non–TNF inhibitor bDMARD or tsDMARD ✓ Switching to a non–TNF inhibitor bDMARD or tsDMARD vs TNF inhibitor |  |  |  |  | ✓ Use combinations of csDMARDs or non TNF biologic or tsDMARDs over TNFi ✓ CHF worsening on current TNFi therapy: Use combination DMARDs on non-TNF or tsDMARDs over another TNFi |  |  |  |  |  |  |  |
| Cardiovascular disease (CVD) |  |  |  |  |  | ✓✓ Risk assessment every 5 years ✓✓ CVD risk prediction models should be adapted  ✓ Screening for asymptomatic atherosclerotic plaques  ✓✓ Lifestyle recommendations  ✓ Antihypertensives and statins  ✓✓ Use caution when prescribing NSAIDs |  |  |  |  |  |  |  |
| Non-alcoholic fatty liver disease | ✓ MTX vs alternative DMARDs |  |  |  |  |  |  |  |  |  |  |  |  |
| Persistent hypogammaglobulinemia without infection | ✓ Rituximab |  |  |  |  |  |  |  |  |  |  |  |  |
| Subcutaneous nodules | ✓ MTX vs alternative DMARDS ✓ Switching to a non-MTX DMARD over continuing MTX |  |  |  |  |  |  |  |  |  |  |  |  |
| Lymphoproliferative disorder | ✓ Rituximab |  |  |  |  |  |  |  |  |  |  |  |  |

✓✓Should do; ✓ Could do; ? Uncertain; O Do not do.

ACR – American College of Rheumatology; APLAR – Asia Pacific League of Associations for Rheumatology; BDMARDs – biologic disease-modifying anti-rheumatic drug; BSR – Brazilian Society of Rheumatology; CRA – Canadian Rheumatology Association; CVD – Cardiovascular disease; csDMARDs – conventional synthetic disease-modifying anti-rheumatic drug; DMARD – disease-modifying anti-rheumatic drug; EULAR – The European League Against Rheumatism; HBV – Hepatitis B virus; HCV – Hepatitis C virus; IGRA – Interferon gamma release assay test; ISR – Italian Society of Rheumatology; MaHTAS – Malaysia Health Technology Assessment Section; NICE – National Institute for Health and Care Excellence; SER – Spanish Rheumatology Society; TB – Tuberculosis; TLAR – Turkish League Against Rheumatism; TNF – Tumor necrosis factor; TNF-i – Tumor necrosis factor inhibitor; tsDMARDs – targeted synthetic disease-modifying antirheumatic drugs; TST – Tuberculin Skin Test.

**Table 4. Surgical recommendations**

|  | ACR (2021) [48] | APLAR [45] | BSR  [42] | CRA  [51] | EULAR  (2023) [50] | ISR  [4] | MaHTAS [43] | NICE [44] | Peter et al. [49] | Santos et al. [47] | SER  [41] | Tenten-Diepenmaat et al. [46] | TLAR  [8] |
| --- | --- | --- | --- | --- | --- | --- | --- | --- | --- | --- | --- | --- | --- |
| Refer for surgical opinion |  |  |  |  |  |  |  | ✓✓ Suspected persistent synovitis of undetermined cause, negative anti-cyclic citrullinated peptide antibodies or rheumatoid factor, the small joints of the hands or feet are affected, > more than one joint is affected, there has been a delay of 3 months or longer between onset of symptoms and seeking medical advice ✓✓ Do not respond to optimal non-surgical management: Persistent pain, worsening joint function, progressive deformity, persistent localised synovitis  ✓ (Imminent or actual tendon rupture, nerve compression, stress fracture) |  |  |  | ✓✓ Do not response to conservative treatment. |  |
| Education on surgical benefits |  |  |  |  |  |  |  | ✓✓ |  |  |  |  |  |
| Medical and surgical management |  |  |  |  |  |  |  | ✓✓ (Suspected or proven septic arthritis) |  |  |  |  |  |
| Magnetic resonance imaging and surgical referral |  |  |  |  |  |  |  | ✓✓ (Cervical myelopathy) |  |  |  |  |  |
| Joint replacement surgery for younger adults |  |  |  |  |  |  |  | ✓ |  |  |  |  |  |
| Lower limb surgical interventions |  |  |  |  |  |  |  |  |  |  |  | ✓ Resection arthroplasty of the MTP joints  ✓ Arthrodesis of the MTP1 joint  ✓✓ Arthrodesis of the subtalar joint and/or arthrodesis of the calcaneocuboid joint and talonavicular joint  ✓ Arthrodesis of the tibiotalar joint or an ankle prosthesis |  |

✓✓Should do; ✓ Could do.

ACR – American College of Rheumatology; APLAR – Asia Pacific League of Associations for Rheumatology; BSR – Brazilian Society of Rheumatology; CRA – Canadian Rheumatology Association; EULAR – The European League Against Rheumatism; ISR – Italian Society of Rheumatology; MaHTAS – Malaysia Health Technology Assessment Section; MTP – Metatarsophalangeal; NICE – National Institute for Health and Care Excellence; SER – Spanish Rheumatology Society; TLAR – Turkish League Against Rheumatism.
