# Supplementary material for: What are the core recommendations for rheumatoid arthritis care? Systematic review of clinical practice guidelines
Source: Clin Rheumatol. 2023 Jun 9;42(9):2267–78. doi: 10.1007/s10067-023-06654-0 (PMC10412487; doi:10.1007/s10067-023-06654-0)
Supplement: Supplementary file 5 — Supplementary file5 (DOCX 187 KB) [file 10067_2023_6654_MOESM5_ESM.docx]

**Online Resource 5. Narrative synthesis of CPG recommendations**

This table is a synthesis of Gout CPG recommendations. A common consensus statement was identified when two or more CPGs reported on an intervention. Consensus statements were either: ‘should do’, ‘could do’, ‘do not do’ or ‘no consensus’. Where one CPG reported on an intervention, no consensus statement was developed (Table 5).

**Table 1. Non-pharmacological management interventions narrative summary**

| **Management interventions** | **Narrative Summary** |
| --- | --- |
| **Patient education** | Six CPGs strongly recommended [41-44, 46, 47] and one CPG conditionally recommended [49] patient education for people with rheumatoid arthritis (RA).  Educational programs [41, 44, 47] and supporting materials [47] should be offered as verbal and written information, that is written in a way that can be easily understood [44]. These programs should be based on a trusting therapeutic relationship between the patient and clinician, to improve patient adherence to treatment modalities [41].  Throughout the course of their disease, clinicians should offer patients the opportunity to talk about and agree all aspects of their care and respect the decisions they make [44]. Information and advice can be individualised for each patient, and include information to support self-management and optimising health and well-being [49].  Content should include:   - General aspects of the disease [47, 49] to improve the patients understanding of RA [44]. - Possible consequences of RA [49]. - Management options [44, 47, 49] - Risks and benefits of treatment options [44]. - Patients’ role in the shared-care process [47]. - Common assessments and interventions [47]. - Countering any misconceptions they may have [44]. - Counselling on lifestyle habits [42]; mentioning the importance of exercise and a healthy lifestyle (including decreasing stress and fatigue) and how this lifestyle can be achieved and maintained [49]. - Information about how and when to access specialist care [44].   The following information should be included for those who experience hand or foot RA-related symptoms:   - Joint protection advice with hand strengthening and mobilisation exercises [43]; - Individual shoe-advice e.g. information on fit, cosmetics, function, durability and correct use of the shoes [46]. - Preventive foot care [46]. - Cause and disease course of RA-related foot disease [46]. - How to recognise infection and increased disease activity (systemic and local) [46]. - Footcare and hygiene [46]. - Recognition and use of adequate footwear [46]. - Encourage a timely consultation by a healthcare professional if sign/symptoms of a foot infection, increased disease activity, pain, problems finding adequate footwear, and skin and nail conditions; and who would be the appropriate healthcare professional [46]. - Treatment plan in relation to footcare [46]. - The importance of treatment adherence and compliance [46]. - The expected treatment outcomes in relation to pain, physical functioning, activities, and participation levels [46]. - Possible adverse events [46]. - Costs and reimbursement of the treatment [46].   Consensus: Patient education should be included as a standard practice of care.  Information can be delivered either as verbally or written and content should include: information on RA, its management options (benefits and risks), common assessments and address patients beliefs about RA and its care. |
| **Patient centered care (PCC)/ Shared decision making** | Eight CPGs strongly recommended care should be patient centered and shared decision making [8, 42, 44-48, 50]. The clinician should provide information and/or clarification about the disease, including the short and long-term outlook of the disease [47] and the available therapeutic options [42, 47], then both patients and clinicians should follow a shared decision-making process [8, 42, 45-48]. Clinicians should respect patients decisions regarding RA care [44].  A treatment option should be started once the patient is diagnosed [45] and should be tailored to the individual patient [46] circumstances. These include: disease activity [8, 50] and/or poor prognosis [45], comorbidities [8, 45, 50], progression of structural damage [8, 50], clinical features, abilities, preferences and needs [47].  Interventions should be targeted to specific individualised goals, regularly monitored with validated instruments and adapted accordingly [47]. Safety of medications [42, 50], individual [8, 50], medical and societal costs should be taken into consideration [8, 42, 50], especially when medications report a similar efficacy [42].  Consensus: Patients and clinicians should adhere to a shared decision-making process and care should be tailored to the patient and their circumstances. |
| **Exercise** | Four CPGs strongly recommended [43, 46, 47, 49] and two CPGs conditionally recommended [44, 46] exercise therapy for people with RA. All patients should follow a regular physical exercise program to reduce pain, functional disability, fatigue and global impact of disease [47]. This included ‘general’ exercise therapy [46, 47], hydro-kinesiotherapy [47], and joint specific programs.  **Hand and wrist programs**  Two CPGs supported hand exercises programs [43, 44]. One CPG recommended a combination of joint protection advice with hand strengthening and mobilisation exercises [43]. Whilst the other CPG supported a tailored strengthening and stretching program, delivered by a practitioner with relevant skills and training [44]. Interestingly, the CPG recommended that an exercise program can be considered if people experience hand or wrist pain and dysfunction and they are not on a drug regimen for RA, or they have been on a stable drug regimen for RA for at least 3 months [44].  **Foot and ankle programs** Exercise therapy specific to the foot and ankle can include: strengthening exercises for the intrinsic foot muscles and M. tibialis posterior, active stretching exercises for the plantar fascia, achilles-tendon, and peroneal muscles and active exercises to improve joint mobility [46].  One CPG recommended that physiotherapists (PTs), classify patients with RA into one of 3 treatment profiles based on the initial assessment [49]:   1. A short period of education, advice, and exercise/movement instruction.   Exercises can be offered and are to be completed primarily independently [49].   1. A short period of guidance and supervision in addition to 1, eg, due to the complexity or severity of problems or limited self-management skills.   Exercise therapy should be offered [49].   1. Intensified guidance and supervision in addition to 1, eg, due to the presence of serious comorbidity or complications of the disease or its treatment.   Exercise therapy can be offered [49].  Regardless of the treatment profile, exercise therapy recommendations should be aligned with the patient’s need for assistance, and adhere to the principles regarding the frequency, intensity, type, and duration of the exercise therapy [49].  Consensus: Exercise therapy should be offered to all patients with RA, and tailored to individual patient circumstances. Modalities can include general exercise therapy, hydro kinesiotherapy and joint specific programs; comprising of strengthening, stretching and joint mobility exercises. |
| **Other non-pharmacological management strategies** | One CPG conditionally recommended non-pharmacological interventions can be included as part of standard care, as an adjunctive or as an alternative to medication or surgery where appropriate [47]; they can only be considered if there is no evidence based supported alternative, if they have consensual approval by qualified health professionals and they are safe and accepted by a duly informed patient [47].  Examples include: pain relief, energy management, joint alignment and support, thermotherapy, psychological interventions, daily leisure and work activities, family involvement, social participation and social care, sleep hygiene and general management of comorbidities [47].  Whilst one CPG recommended against offering low-level laser therapy, electrostimulation (including transcutaneous electrical nerve stimulation), ultrasound, massage, thermotherapy, medical taping, dry needling and passive mobilisations of joints and muscles [49]. They conditionally recommended for short-term passive mobilisation of an affected joint only as an adjunct to exercise therapy for patients without active inflammation to increase joint mobility and recommended against for patients with cervical problems [49].  Consensus: No consensus. |
| **Shoes** | |
| **Ready-made therapeutic shoes** | One CPG strongly recommended and one CPG conditionally recommended therapeutic footwear for patients with RA if indicated [44, 46]. One CPG recommended ready-made therapeutic shoes in cases of abnormal foot function, foot joint damage/deformity, or malalignment of the feet, and their feet that do not fit in over-the-counter shoes, but for whom custom-made shoes are not indicated [46]. These shoes should have extra depth, support, incorporated inlays, and optional technical adaptation can reduce forefoot plantar pressure and foot pain and improve gait characteristics, physical functioning, and health-related quality of life [46].  Consensus: Ready-made therapeutic shoes can be considered for patients with RA. |
| **Orthoses and Braces** | |
| **Orthoses** | Three CPGs strongly recommended foot orthoses [46, 47]/functional insoles [44] for people with RA with abnormal foot function, when adequate over-the-counter shoes are insufficient in reducing foot symptoms such as: forefoot plantar pressure and pain. The function of foot orthoses should be assessed in relation to the patient’s footwear [46]. Foot orthoses can reduce pain, functional disability and global impact of disease [47].  Consensus: Orthoses should be offered to patients with RA, and abnormal foot function, where over the counter shoes have been insufficient in symptom relief. |
| **Health professionals** | |
| **Multi-disciplinary team** | Five CPGs strongly recommended [8, 42, 44, 46, 47] and one conditionally recommended [43] patients should have a multi-disciplinary team [42, 44, 46, 47], with a named member who is responsible for coordinating their care [44]. The team should:   - Conduct regular assessments of the patients pain levels, fatigue, impact on daily life activities, mobility, ability to work or take part in social or leisure activities, quality of life, mood and impact on sexual relationships [44]. - Provide relevant information for the patient to be involved in the shared decision-making process, including the disease prognosis and of the available scope of treatment interventions [47] (non-pharmacological and pharmacological); adopting a holistic bio-psycho-social view to health [47]. - Select interventions relevant to the patient and encourage adherence [47]. - Focus on prevention and management of comorbidities and adverse effects [47]. - Patients should be regularly inquired for unmet needs, using validated large-scope instruments, and referenced to the most appropriate health professional(s) in the team [47].   A multi-disciplinary team approach is needed to diagnose and manage RA-related foot problems, as this often requires the expertise of several disciplines [46]. One CPG recommended that rheumatologists, physical medicine and rehabilitation specialists are the main clinicians responsible for RA care [8].  Consensus: All patients should be offered a multi-disciplinary team approach to care. |
| **Nursing** | Four CPGs strongly recommended nurses [41, 43, 44, 46], who specialised in rheumatology are included in the care team [46].  Nursing staff can lead coordination of the care [43, 44], deliver ongoing, specific individual or group educational programmes [41] and/or perform regular feet check-ups [46]. Check-ups should include a patient history of foot disease, foot inspection, and palpation of foot joints for the detection of swelling and pain at a minimum [46].  Consensus: Nursing staff should be included in the multi-disciplinary team. Their role includes coordinating patient care, delivering education programs and performing regular feet check-ups if relevant. |
| **Physiotherapy** | Two CPGs strongly recommended PTs should be offered to all patients with RA [44, 49] with periodic review [44].  Their role is to encourage improving general fitness [44] and exercise therapy [44, 49] with joint flexibility and muscle strengthening exercises and managing other functional impairments [44]. Education of the short-term pain relief of passive treatment modalities such as transcutaneous electrical nerve stimulators should be provided to patients [44].  PTs should perform a comprehensive subjective assessment of the patient’s health status, the course of the disease and its current impact on the patient’s life and previous and current pharmacological treatment [49]. They should then perform an objective assessment exploring current disease activity (extent and severity of joint pain, swelling, and limited joint ROM), the presence of structural joint damage and deformities, general exercise tolerance, and muscle function during physical examination, including examination of the cervical spine and the jaw joints [49]. PT should use measurement tools to assist with diagnosis and evaluating treatment, it is recommended to combine a self-reported questionnaire and a performance based test to examine physical functioning [49].  The following are examples of outcome measures that should be used [49]:   - Numeric Rating Scale for fatigue - Numeric Pain Rating Scale - Borg Rating of Perceived Exertion Scale (Borg RPE scale 6-20) - Dutch Consensus Health Assessment Questionnaire Disability Index - Patient-Specific Complaints - Six-Minute Walk Test   Consensus: Physical therapy should be offered to all patients, PTs can assist with history taking, assessment and treatment of RA. The main treatment modality that should be encouraged is exercise therapy. |
| **Psychological** | Two CPGs strongly recommended offering psychological interventions to patients with RA [44, 47]. Intervention options include: relaxation, stress management and cognitive coping skills (such as managing negative thoughts) [44]. The aim of these is to assist with adjustment to living with their condition [44], help reduce pain, functional disability, fatigue and global impact of disease [47].  Consensus: Psychological interventions should be offered to patients with RA. |
| **Rheumatologist** | Six CPGs strongly recommended that a rheumatologist is involved [8, 42-44, 46, 50] in a multi-disciplinary team [42], either as the primary care provider [8, 42] or a co-management plan with healthcare providers [8, 43]. All patients with RA should have rapid access to specialist care [44].  Two CPGs discussed indications for referral to rheumatologists [43, 44], these included:   - All patients suspected of having RA [43]. - Suspected persistent synovitis of undetermined cause [44]. - Refer urgently (even with a normal acute-phase response, negative anti-cyclic citrullinated peptide [CCP] antibodies or rheumatoid factor) if:   - The small joints of the hands or feet are affected   - More than one joint is affected   - A delay of 3 months or longer between onset of symptoms and seeking medical advice [44].   Rheumatologists should perform regular feet check-ups [46]. Check-ups should include a patient history of foot disease, foot inspection, and palpation of foot joints for the detection of swelling and pain at a minimum [46].  Consensus: Rheumatologists should be considered in a multi-disciplinary team or as the primary care provider for patients with RA. |

CPGs – Clinical practice guidelines; PCC – Patient centered care; PT – Physiotherapy/Physical therapy; RA – Rheumatoid arthritis; RPE – Rating of perceived exertion.

**Table 2. Pharmacological management interventions narrative summary**

| **Management Principles** | **Narrative Summary** |
| --- | --- |
| **Pharmacological management** | |
| **Early initiation of DMARDs** | Two CPGs strongly recommended Disease-Modifying Antirheumatic Drug **(**DMARD) therapy is initiated ‘soon after’ RA diagnosis is made [8, 50].  Consensus: DMARD therapy should begin once a patient is diagnosed with RA. |
| **Initial treatment with csDMARDs** | Eight CPGs strongly recommended Conventional Synthetic DMARDs (csDMARDs) [4, 8, 41-45, 50] and one CPG reported a combination of both strong and conditional recommendations [48].  csDMARDs should begin as soon as a person is diagnosed with RA [4, 42, 45], within 3 months of onset of persistent symptoms [44]. Majority of CPGs recommended that monotherapy should be offered in the first instance [41, 45].  Consensus: csDMARDs should be initiated when a patient is diagnosed with RA. |
| **Selection of csDMARDs** | **Methotrexate (MTX)**  Eight CPGs strongly recommended MTX [4, 8, 41-45, 50], whilst one CPG report a combination of strong and conditional recommendations depending on the context for people with RA [48].  MTX should be used as a first-choice treatment strategy csDMARD [4, 42, 43, 45, 50], unless contraindicated [43-45]. Dosages varied amongst CPGs from at least 10 mg/week [41] (when in combination with anti-TNF agents) to at least 15 mg/week within 4 to 6 weeks [48]. Preferred method was in an oral form, before considering a split dose of oral MTX over 24 hours or subcutaneous injections and/or an increased dose of folic/folinic acid rather than switching to alternative DMARD(s) [48].  MTX monotherapy is recommended over other pharmacological options, for example: other csDMARDs (hydroxychloroquine (HCQ), Leflunomide (LEF), sulfasalazine (SSZ), bDMARD or tsDMARD monotherapy tumour necrosis factor inhibitors (TNFi), Tofacitinib [48] or combination therapies, such as: MTX, plus a TNFi, non–TNF inhibitor, biologic DMARDs (bDMARD) targeted synthetic (tsDMARD), or double or triple csDMARD therapy [48]. Combination therapy (csDMARDs or a bDMARD) can be considered when monotherapy fails [41], despite an escalated dose (as tolerated) [44].  Consensus: Methotrexate monotherapy should be used as a first-choice treatment strategy csDMARD.  **LEF, SSZ or HCQ**  Five CPGs strongly recommended [4, 8, 41, 42, 44, 50] and three CPGs conditionally recommended [44, 45, 48] LEF, SSZ or HCQ for patients with RA.  LEF, SSZ or HCQ should be added in combination with MTX, for patients who haven’t achieved treatment target [42, 44].  LEF [4, 8, 41, 42, 45, 50] (with a biologic [41]), SSZ [4, 8, 42, 45, 50] or HCQ [44, 45] should replace MTX, for patients who are contraindicated, exhibit an early intolerance to MTX.  Consensus: LEF, SSZ or Hydroxychloroquine should be considered when a patient is contraindicated, demonstrates an early intolerance to or has failed to achieve treatment target with MTX. |
| **Treatment if csDMARD fails** | **Combination therapy**  One CPG strongly recommended patients have access to several drugs with different modes of action to address the heterogeneity of RA; they may require multiple successive therapies throughout life [50].  Five CPGs strongly recommended [4, 8, 44, 45, 50] and two CPGs conditionally recommended [41, 42] combination therapy of csDMARDs (e.g. double or triple csDMARD [41, 42, 44, 45]) if treatment target is not reached with the first treatment regimen [4, 8, 44, 45], despite dose escalation [44] and in the absence of poor prognostic factors [4, 8, 44, 50] or as first-line therapy [42]. Interestingly, one CPG disagreed, and recommended for patients with poor prognostic factors [45]. MTX should be used an anchor drug, unless contraindicated [42, 45].  One CPG recommended against triple therapy initially, unless Glucocorticoids (GCs) are contraindicated [41].  Combination therapy of csDMARDs should be considered if treatment target is not achieved with csDMARD monotherapy.  **bDMARD or tsDMARDs**  Three CPGs strongly recommended [4, 8, 50], one CPG conditionally recommended [48] and four strongly and conditionally recommended bDMARDs and tsDMARDs [41-43, 45] depending on the context.  A bDMARD and/or tsDMARD addition should be considered [4, 8, 41, 43, 50] if treatment goal cannot be reached with the first treatment regimen [8] or two schemes of csDMARDs [42] and in the presence of poor prognostic factors [4, 8, 50]. Majority of CPGs recommended this is in conjunction with a csDMARD [8, 41, 42, 45, 50], preferably MTX [42, 45]. Adding a bDMARD or tsDMARD can be recommended over triple therapy if maximum dose of MTX is currently being taken [48].  bDMARDs should be recommended in the first instance [8, 28, 42, 43, 50] with no specific bDMARD being preferred over another [41]; clinicians should consider drug cost and safety [42]. Early bDMARD use can be considered in patients who have active disease with poor prognostic factors [45].  If adding a bDMARD or tsDMARD fails to achieve treatment target this should be swapped to another bDMARD or tsDMARD [4, 8, 42, 45, 48, 50] of the same class [42] or with another mechanism of action is effective and safe [42, 48].  Consensus: bDMARD and tsDMARDs should be recommended if csDMARDs fail to achieve treatment target, with a bDMARD being added in the first instance.  **Janus kinase inhibitor (JKI)**  Five CPGs conditionally recommended for the use of JKI [4, 41, 42, 45, 50]. This can be added to a bsDMARD and tsDMARD if csDMARDs are contraindicated [4, 41] but pertinent risk factors* must be taken into account [50]. Tofacitinib can be used after failure of bDMARD [42, 45].  Consensus: JKI can be recommended in combination with bDMARDs and tsDMARDs if csDMARDs are contraindicated. Tofacitinib can be used after failure of bDMARD.  **Tumor necrosis factor inhibitor (TNF-i)**  Four CPGs conditionally recommended a TNF-i [4, 8, 42, 50]. A TNF-i can be added to a bDMARD or tsDMARD [4]. If a TNF-i fails, another TNF-i or medication can be added of the same class [42] or with a different mechanism [4, 8, 42, 50].  Consensus: TNF-i can be recommended in conjunction with DMARD therapy.  **Non-TNF**  Two CPGs conditionally recommended non-TNF [4, 41] in conjunction with MTX [41] or a bDMARD or tsDMARD [4]. If the first non-TNF fails, a second non-TNF agent or a biologic acting on a different therapeutic target, depending on the type of inefficacy and patient characteristics can be added [41].  Consensus: non-TNF can be recommended in conjunction with DMARD therapy.  **IL-6 inhibitors**  Two CPGs conditionally recommended [8, 50] and one CPG strongly recommended [41] for IL-6 inhibitors. If bDMARDs fail, addition of IL-6 inhibitors or tsDMARDs could be preferred over addition of other bDMARDs [8, 50]. One CPG recommended monotherapy of IL-6 inhibitors rather than an anti-TNF agent if csDMARDs are contraindicated [41].  Consensus: IL-6 inhibitors can be recommended if bDMARDs fail. |
| **Treatment Targets** | **Treatment goal and** **Treat-to-target (T2T) strategy**  Treatment should include both non-pharmacological and pharmacological interventions [47] and focus on assisting patients in maximising their overall quality and enjoyment of life [45, 47], through optimized control of the impact of disease [47] and maintaining physical functioning [45]. A treat-to-target strategy is recommended rather than a non-targeted approach [43, 44, 48].  Six CPGs strongly recommended that the treatment goal should be sustained clinical remission or if that is not possible, low disease activity [8, 42-45, 50] within six months of using a treat-to-target strategy [43].  One CPG reported a combination of strong and conditional recommendations depending on the context [48]. Remission can be preferred over low disease activity if a patient has increased risk of radiological progression (presence of anti- cyclic citrullinated peptide antibodies or erosions on x-ray at baseline assessment) [44]. Whilst one CPG argues that low disease activity can be preferred over a goal of remission initially [48].  Consensus: Treatment should be initiated early and aim to improve the patient’s quality of life and reduce the impact of disease.  The treatment goal should be remission, or if that is not possible than low disease activity and treatment should follow a treat-to-target strategy. |
| **Monitoring** | **Frequency**  Five CPGs reported on monitoring medications [44, 45, 47, 48, 50].  One CPG recommended treatment is monitored ongoing [44], whilst other CPGs provided time intervals for monitoring based on disease activity. Three CPGs recommended patients should be monitored for disease activity every 1–3 months [45, 50] or within 3 months [48] after initiating treatment or changing treatments until disease is stabilised and treatment target is achieved [45].  If treatment fails to improve the patient’s condition in the first 3 months [50], or achieve treatment target by 6 months, therapy should be adjusted [44, 50]. If treatment is stabilised, and target is achieved patients can be monitored every 3-6months [45]. A review appointment should occur at the 6 months post-achieving treatment target to ensure this is maintained [44].  **Assessment/outcome measures**  A suitable and practical standardised [45] validated measure [47] of disease activity should be routinely performed to assess patients’ response to treatment [45]. The following parameters should be used: Safety monitoring for patients who are on bDMARD therapy [45] /ongoing drug monitoring [44].  Examples of validated instruments described within CPGs main text and included:   - Disease Activity Score 28 joints (DAS28) [4, 8, 43-45] - Simplified Disease Activity Index (SDAI) [4, 8, 41, 43, 45] - Clinical Disease Activity Index (CDAI) [4, 8, 41, 43, 45] - Other criteria e,g, ACR-EULAR criteria [42, 43, 45, 50] - According to a composite measure with no swollen joints [51] - Several CPGs didn’t describe preferred instrument/define remission/low disease activity [46-49].   One CPG recommended monthly monitoring of disease activity using a outcome measure, in addition to measuring C-reactive protein until the patient has achieved treatment target [44].    In those who have achieved a treatment target, offer an annual review to:   - Check for the development of comorbidities, such as hypertension, ischaemic heart disease, osteoporosis and depression [44]. - Assess disease activity and damage, and measure functional ability (using, for example, the Health Assessment Questionnaire [HAQ]) [44]. - Assess symptoms that suggest complications, such as vasculitis and disease of the cervical spine, lung or eye [44]. - Refer to appropriate members of a multidisciplinary team [44]. - Assess the need for referral for surgery [44]. - Assess the effect the disease is having on a person's life [44].   Ultrasound should be avoided in routine monitoring [44].  Consensus: Disease activity should be monitored every 1-3 months if the patient is recently diagnosed or changing treatment strategies. Then monitoring can occur at every 3-6 months if disease is stable and the patient has achieved treatment target, with a review appointment at 6 months.  This should include a practical, standardised and validated disease activity measure. |
| **Tapering** | One CPG strongly recommended [41] and five CPGs conditionally recommended [8, 42, 44, 48, 50] and two CPGs reported both strongly recommended and conditionally recommended [4, 45] taping medications when a patient is in remission. One CPG recommended against tapering if the patient doesn’t have rapid access to care or will experience difficulty re-establishing access to medications [51].  CPGs vary on the amount of time a patient needs to be in remission or at low disease activity before tapering can occur. Several CPGs support 6 months [41, 45, 48, 51], whilst others supported 12 months [44, 45]; One CPG supported both time intervals depending on the treatment being tapered, e.g. corticosteriods at 6 months, bDMARDs at 12 months [45]. The remaining CPGs didn’t provide a time frame [4, 8, 42, 50]. If a treatment target is not met, patients should return promptly to the previous DMARD [44].  Recommendations on medications that can be tapered include:  GCs   - GCs (e.g. Corticosteroids and non-steroidal anti-inflammatories (NSAIDs) [45]) should be tapered [8] in the first instance [50] with the aim of eventually stopping these treatments [45].   DMARD   - Tapering of DMARDs can be considered [44, 45, 50] following glucocorticoid tapering and maintain the treatment target for 6-12months [44, 45]. Cautiously reducing drug doses or stopping drugs in a step-down strategy is recommended [44]. - Whilst on CPG reports continuing all DMARDs at their current dose can be recommended rather than reducing their dosages. A dose reduction can be recommended rather than gradual discontinuation and a gradual discontinuation can be recommended over an abrupt discontinuation for patients who are at target for at least 6 months [48]   bDMARDs   - Progressive taping of bDMARDs [4, 41, 45, 50, 51] can be considered especially if this this treatment is combined with a csDMARD [4, 8]. This may involve reducing [41, 42] or spacing out the doses [42] of the medication and should be recommended despite the risk of relapse [41]. Based on shared-decision making and provided patients are able to rapidly access rheumatology care and re-establish their medications in case of a flare [51].   Based on shared-decision making and provided patients are able to rapidly access rheumatology care and re-establish their medications in case of a flare [51].  tsDMARDs   - tsDMARDs tapering could be considered [4, 50, 51] especially when in conjunction with csDMARDs [4]. Based on shared-decision making and provided patients are able to rapidly access rheumatology care and re-establish their medications in case of a flare [51].   csDMARDs   - If a patient is in persistent remission, tapering the csDMARD could be considered [4, 8, 45, 50] with caution and as a shared decision between the patient and clinician [45]. - Gradual discontinuation of SSZ can be recommended over HCQ for people taking triple therapy who wish to discontinue a DMARD [48]. - Gradual discontinuation of MTX can be recommended over bDMARD or tsDMARD for patients taking MTX + a bDMARD or tsDMARD [48].   Consensus: No consensus |
| **Adjunctive therapy** | **NSAIDs**  One CPG strongly recommended [45] and two CPGs conditionally recommended NSAIDs [43, 44] including traditional NSAIDs (+/- a proton pump inhibitor or cox II selective inhibitors [44, 45]. This should be taken orally [8, 44], at the lowest effective dose for the shortest duration [44, 45] in combination with DMARDs [43] to reduce pain and inflammation [8, 43].  Clinicians should consider gastrointestinal, liver and cardio-renal toxicity, and the person's risk factors, including age and pregnancy and monitor these risk factors for adverse events on a regular basis [44]. If a person with RA needs to take low-dose aspirin, other treatments should be considered before adding an NSAID with a proton pump inhibitor [44].  Consensus: NSAIDs can be used as adjunctive treatment at the lowest effective dose for the shortest duration.  **GCs e.g. Prednisolone**  Four CPGs strongly recommended [8, 41, 42], four CPGs conditionally recommended [43, 44, 46, 48, 50] GCs and two CPGs reported a either a combination of recommendations depending on context [4, 45]. Glucocorticoids can rapidly decrease inflammation [44] or reduce radiographic progression [45].  Short-term glucocorticoids can be considered:   - In response to a patient experiencing a flare/to control active RA [4, 44]. - In combination when initiating or changing csDMARDs [4, 8, 41, 43-45, 50]. - and the risk-benefit ratio have been considered [42].   Monotherapy is not recommended [45].  GCs can include different dose regimens and routes of administration [8, 50] e.g. oral, intramuscular, or intra-articular injections [44]. Injections could be considered for the relief of local symptoms of inflammation [4, 46], pain, synovitis and/or tendinitis (in the foot) and could be guided by ultrasonography to improve accuracy [46].  The chosen GCs should be administered at the lowest dose [4, 42-45] and tapered as rapidly as clinically feasible [4, 8, 41, 42, 45, 50] to avoid side effects [4, 8]. Definitions of short-term varied amongst CPGs from <3 months [43] to <6 months [4]. Similarly, dosage of prednisone, a commonly selected GCs ranged from ≤ 7.5 [8, 45], ≤10 mg once daily [43] or 10-30 mg/day [41] and is recommended in combination with a csDMARDs [41]. Long-term treatment with GCs can be prescribed when patients are aware of the complications and have been offered other treatment options [44]. The adding or/switching of DMARDs is recommended over continuing GCs [48].  Consensus: Short-term GCs can be considered in combination with other interventions, at a low dose and tapered as soon as clinically feasible. |
| **Pre-treatment investigations and vaccinations** | |
| **Pre-treatment investigations** | Two CPGs strongly recommended that patients with RA should undergo investigations prior to starting medications [43, 45] e.g. bDMARDs, tsDMARDs [43] and csDMARDS [45].  Patients should complete a blood count [45] and be examined for active or current infections [45] and viruses including: Hepatitis B (HBV) and Hepatitis C (HCV) infection [43, 45], tuberculosis [43, 45] and human immunodeficiency virus [43].  Patients should also be assessed for comorbidities and possible contraindications to treatment modalities including: screening for tumours and malignancies, renal and liver function tests, chest radiography and pregnancy screening [45].  Consensus: Patients should undergo investigations before commencing treatment. These can include blood tests, renal and liver function tests. |
| **Vaccinations** | Three CPGs reported recommendations strongly for or against in relation to vaccinations [4, 42, 45].  Patients vaccination status and special situations such as pregnancy and lactation should be recorded [45]. Vaccination records should be updated as needed [42].   - Ideally all vaccinations should be administered four weeks prior to initiating therapy (bDMARD [4, 45] or tsDMARDs [4]); especially live vaccines [45]. - Concurrent live, attenuated vaccines are contraindications for patients being treated with bDMARD [4, 45] or tsDMARDs [4]. - Killed vaccines (Pneumococcal, Influenza- yearly, HBV) or recombinant vaccines (Human Papilloma) should be given before initiating or during DMARD therapy [4].   Consensus: All patients should be assessed for infections and vaccinations should be provided prior to DMARD therapy and updated as needed.  Do not administer live vaccines whilst patients being treated with bDMARDs or tsDMARDs. |

bDMARD – biologic disease-modifying anti-rheumatic drug; CDAI – Clinical Disease Activity Index; CHF – congestive heart failure; CPG – clinical practice guideline; csDMARD – conventional synthetic disease-modifying anti-rheumatic drug; DAS28 – Disease Activity Score 28 joints; DMARD – disease-modifying anti-rheumatic drug; GC – Glucocorticoids; HAQ – Health Assessment Questionnaire; HBV – Hepatitis B virus; HCQ – hydroxychloroquine; HCV – Hepatitis C virus; LEF – Leflunomide; MTX – methotrexate; NSAID – Non-steroidal anti-inflammatories; RA – Rheumatoid arthritis; SDAI – Simplified Disease Activity Index; SSZ – sulfasalazine; TNFi – tumour necrosis factor inhibitors; tsDMARDs – targeted synthetic disease-modifying anti-rheumatic drug.

**Table 3. Special populations narrative summary**

| **Special populations** | |
| --- | --- |
| **Serious Infections** | Three clinical practice guidelines (CPGs) reported on infections with varying recommendations on the preferred treatment [41, 45, 48]. One CPG strongly recommended either with abatacept or if an anti-tumour necrosis factor (TNF) is preferred, etanercept if a patient is on biologic disease-modifying anti-rheumatic drug (bDMARD) therapy and has developed a serious infection [41]. Whilst another CPG disagrees and advises bDMARDs should be stopped and the patient be treated appropriately if experiencing an active infection of an infection is suspected [45].  One CPG conditionally recommended that if the patient has experienced a serious infection in the last 12 months adding a conventional synthetic disease-modifying anti-rheumatic drug (csDMARD) is preferred rather than a bDMARD or targeted synthetic disease-modifying anti-rheumatic drug (tsDMARD), although noted that disease-modifying anti-rheumatic drug (DMARDs) can be added or switched before considering initiation/dose escalation of glucocorticoids [48].  Consensus: No consensus. |
| **Cancer** | Two CPGs reported on interventions used for patients with cancer and rheumatoid arthritis (RA) [4, 41].  One CPG strongly recommended that patients who have a history of cancer are assessed on an individual basis before starting bDMARDs, with no specific treatment being preferred over another [41]. Treatment decisions should be made based on consensus between the patient, oncologist and other specialists involved [41].  One CPG reported specific recommendations for certain types of cancers:  Previously been treated for low-grade melanoma or non-melanoma skin cancer:   - csDMARDs should be recommended rather than tsDMARDs or bDMARDs, although bDMARDs and tsDMARDs can be recommended with close skin surveillance by a dermatologist [4].   Previously treated for lymphoproliferative disorder:   - A combination of csDMARDs or abatacept or tocilizumab could be recommended over TNF-inhibitor - Rituximab should be recommended over TNF-inhibitor [4].   Previously treated solid organ malignancy   - They should be treated consistent with recommendations for people with RA and no malignancy [4].   Consensus: No consensus. |
| **Tuberculosis (TB)** | Two CPGs strongly recommended that patients are screened for TB prior to commencing bDMARD [4, 45] or tsDMARD therapy [4], this should include The Mantoux tuberculin skin test (TST) or Interferon gamma release assay test (IGRA). IGRA being preferred if the patient has a history of Bacillus Calmette-Guerin vaccination [4]. Annual testing is recommended in RA patients who live, travel or work in situations where TB exposure is likely while they continue treatment with bDMARDs or tsDMARDs [4].  If the patient returns a negative result for TST or IGRA, exhibits no risk factors and/or clinical suspicion for TB they may not require additional testing [4]. However, if the patient returns a positive result on either TST or IGRA initially, they should repeat the screening and complete a chest radiograph [4]. If the patient is positive for past TB exposure or active TB, then they should complete a sputum examination [4].  If the RA patient has active or latent TB based on the test results they should be prescribed appropriate anti-tubercular treatment [4, 45] and a referral to a specialist (pulmonologist or infectious disease specialist) can be considered [4]. Those with active TB need to be adequately treated [45] and experiencing one month of latent TB with anti-tubercular medications before initiating or resuming bDMARD or tsDMARDs therapy [4].  Consensus: All patients should be screened for TB infection before commencing DMARD therapy. If the patient has active or latent TB they should be prescribed anti-tubercular treatment and be adequately treated before resuming bDMARDs or tsDMARDs. |
| **Hepatitis** | Two CPGs strongly recommended that patients should be screened for Hepatitis B virus (HBV) infections [45] (e.g. HBsAg, antiHBcAb) and immunity (e.g. antiHBsAb) [4] and Hepatitis C virus (HCV) before commencing bDMARD [4, 45] or tsDMARD therapy [4].  Three CPGs reported a mix of strong, conditional and do not do recommendations for treatment options in HBV or HCV patients [4, 45, 48].  **HBV**   - If a patient has an active or untreated chronic HBV infection, bDMARDs should not be recommended [45]. - AntiHBcAb positive, HBsAg negative   - Further examination: HBV, DNA, liver function tests before starting immunosuppressive therapy [4].   - Viral load monitoring every 6-12months [4], frequent monitoring can be recommended over prophylactic antiviral therapy for patients initiating a bDMARD other than rituximab or a tsDMARD [48].   - Treatment consistent with recommendations for RA patients who are negative for HBV [4], whereas one CPG recommended prophylactic antiviral therapy should be recommended over frequent monitoring alone for patients initiating rituximab who are AntiHBcAb positive (regardless of hepatitis B surface antigen status) [48]. - HBsAg positive   - Further examination: quantitative HBsAg, HBeAg, antiHBe, HBV DNA and anti HDV IgG and liver function tests, before starting immunosuppressive therapy [4].   - Prophylactic antiviral therapy should be recommended over frequent monitoring alone for patients initiating any bDMARD or tsDMARD who are Anti-HBcAb positive and HBsAg positive [48]. - Active HBV carriers   - Entecavir or tenofovir should be recommended before starting immunosuppressive therapy [4]. - Acute HB occurring in patients with RA, such as asymptomatic infections occurring in patients previously negative for HBV serology   - Antiviral treatment should be recommended consistent with international guidelines [4]. - In inactive HBV carriers   - Prophylaxis should be started 4 weeks before the immunosuppressive therapy and continued for 12 months after its discontinuation (24 months in the case of rituximab-treated patients) [4].   - Patients stopping prophylaxis should be closely monitored [4].   **HCV**   - If a patient has an active HCV infection:   - bDMARDs should not be recommended [45]   - Further investigations: gastroenterological / infectious evaluation for any anti-viral eradicative therapy and should not be treated differently from patients with AR without HCV infection [4].   Consensus: Patients should be screened for HBV or HBC infections and receive further evaluations and treatment based on their results. |
| **Congestive heart failure (CHF)** | Two CPGs conditionally recommended interventions with patients who have CHF [4, 48].  Use combination of DMARDS (csDMARDs, bDMARDs or tsDMARDs), and a non-TNF-inhibitor e.g. tofacitinib over a TNF-inhibitor [4, 48].  Even in cases of heart failure, the addition of or switching to a non–TNF inhibitor, bDMARD or tsDMARD is still preferred over the addition of or continuing a TNF inhibitor for patients have an inadequate response to csDMARDs [48]. A TNF-inhibitor should only be used if there are no other reasonable options, and then, perhaps, only in compensated heart failure [4].  Consensus: Do not use a TNF inhibitor in persons with a history of CHF, unless there is no other reasonable option, and the CHF is compensated. |

bDMARD – biologic disease-modifying anti-rheumatic drug; CHF – Congestive heart failure; CPG – clinical practice guideline; csDMARD – conventional synthetic disease-modifying anti-rheumatic drug; DMARD – disease-modifying anti-rheumatic drug; HBV – Hepatitis B virus (HBV); HCV – Hepatitis C virus (HCV); IGRA – Interferon gamma release assay test; RA – Rheumatoid arthritis; TB – Tuberculosis; TNF – tumour necrosis factor; tsDMARDs – targeted synthetic disease-modifying anti-rheumatic drug; TST – The Mantoux tuberculin skin test.

**Table 4. Surgical management interventions narrative summary**

| **Management Principles** | **Narrative Summary** |
| --- | --- |
| **Referral for surgical opinion** | Two CPGs strongly recommend early consultation with an orthopaedic surgeon [46] or specialist surgical opinion [44], in certain clinical situations.  If any of the following do not respond to optimal non-surgical management:   - Persistent pain [44, 46] due to joint damage or other identifiable soft tissue cause [44]. - Worsening joint function [44] or stiffness [46]. - Progressive deformity [44] malalignment of the foot (e.g., hammer toes) causing mobility limitations and pain or problems finding adequate shoes [46]. - Persistent localised synovitis [44] > 6 months of synovitis in foot and ankle joints [46]. - Tenosynovitis or tendon ruptures [46] imminent or actual tendon rupture [44]. - Returning callosity/clavus [46]. - Wounds/(pre)ulcers [46]. - Osteomyelitis/septic arthritis [46]. - Nerve compression [44]. - Stress fracture [44].   This aims to address joint damage or deformity before it becomes irreversible and provide pain relief [44]. Cosmetic improvements should not be priority [44].  Referral should be urgent if the patient the patient has suspected persistent synovitis of undetermined cause, (even with a normal acute-phase response, negative anti-cyclic citrullinated peptide antibodies or rheumatoid factor) and any of the following applies:   - the small joints of the hands or feet are affected - more than one joint is affected - there has been a delay of 3 months or longer between onset of symptoms and seeking medical advice [44].   Consensus: Surgery should be considered when conservative management has not been successful, and the patient meets the above criteria. |

CPG – Clinical practice guideline.

**Table 5. Single CPG recommendations on interventions**

| **Non-pharmacological**  One clinical practice guideline (CPG) strongly recommended that non-pharmacological interventions are an important aspect of standard care and should be considered throughout the course of the disease as an adjunctive or alternative to pharmacological or surgical interventions where appropriate [47]. Examples of interventions include: joint alignment and support, exercise, education, sleep and energy management [47].. |
| --- |
| **Dietary advice and complementary therapies**  One CPG conditionally recommended Mediterranean diet principles e.g. more bread, fruit, vegetables, and fish; less meat; and replace butter and cheese with products based on vegetable and plant oils [44]. Clinicians should educate patients that there is no strong evidence to support experimental diets, and therefore should not be considered as an alternative to evidence-based therapies [44]. Diets may provide short-term symptomatic benefit however their long-term efficacy is yet to be determined [44].  One CPG recommended against patients replacing evidence based treatment for complementary therapies and that if patients decided to use these therapies it should not prejudice attitudes of clinicians[44]. |
| **Shoes** |
| **Over-the counter shoes**  One CPG strongly recommended over-the counter shoes for people with rheumatoid arthritis (RA) should have sufficient room in the toe box and a stiff sole allowing a heel-to-toe gait [46].  The following features were conditionally recommended for certain foot conditions and depending on the patient’s wishes:   - Light weight, spacious, adjustable, and easy to close in-step/heel girth. - Strong, raised, and padded heel part. - Inflection points at the metatarsophalangeal (MTP) joints. - Adequate length and width, measured in standing position. - No seams on the inside.   Technical adaptations to over-the-counter shoes can reduce pain and improve physical functioning. These adaptations can be prescribed in patients with abnormal foot function, foot joint damage/deformity, or malalignment of the feet, provided that the feet fit in over-the-counter shoes [46].  **Custom-made therapeutic shoes**  One CPG conditionally recommended custom-made therapeutic shoes for patients with abnormal foot function, foot joint damage/deformity, or malalignment of the feet, and feet that do not fit in over-the-counter shoes or ready-made therapeutic shoes [46].  These shoes should be worn all day after a habituation period and can reduce pain and improve physical functioning [46]. |
| **Orthoses and Braces** |
| **Rigid foot orthoses; Total contact foot orthoses**  One CPG strongly recommended rigid foot orthoses for feet with correctable malalignment, to assist with foot positioning during weight-bearing. Whereas if the feet have malalignment that can’t be corrected or fragile skin, total contact foot orthoses are strongly recommended. The material used for the production of total contact foot orthoses depends on the required characteristics of the foot orthoses [46].  **Silicone toe orthosis; Toenail brace**  One CPG conditionally recommended for a silicone toe orthosis in cases of malalignment of toes and secondary pain or high pressure. This can be prescribed if there is a sensibility disorder or peripheral artery disease; a skin defect on the foot of interest; and sufficient room in the shoe for wearing the toe orthosis. Whilst a toenail brace was conditionally recommended for treating ingrowing or an ingrown toenail if the patient has a sensibility disorder or peripheral artery disease; a skin defect, inflammation, or onycholysis on the toe of interest; and the use of biologicals [46]. |
| **Health professionals** |
| **Occupational therapy**  One CPG strongly recommended specialist occupational therapy involvement in care, with regular review if they are experiencing difficulties with any of their everyday activities, or problems with hand function [44].  **Podiatry**  One CPG strongly recommended all patients with RA and foot problems should have podiatrist involvement in the care team for assessment and regular review of their foot health needs [44].  **Supervised program**  One CPG conditionally recommended a supervised treatment program to improve adherence, especially in women, elderly and comorbid patients [41]. |
| **Pharmacological**  One CPG strongly recommended pharmacological interventions are considered as part of standard care [47]. |
| **Anti-IL-6**  One CPG strongly recommended the use of an anti-IL6 agent rather than an anti- TNF agent as monotherapy [41].  **Anakira**  One CPG recommended against the use of anakinra except in the context of a controlled, long-term clinical study and against the use of Anakira in combination with tumour necrosis factor-α (TNF-α) inhibitor therapy [44].  Patients taking anakinra should continue therapy until they and their health professional recommends stopping to avoid impacting on the patients wellbeing [44]. |
| **Recommendations for specific populations** |
| **Interstitial lung disease**  One CPG strongly recommended abatacept for patients with RA and interstitial lung disease who require treatment with a biologic, whilst conditionally recommended rituximab as an alternative [41].  Based on current available evidence, the CPG was unable to determine either for or against these medications for interstitial pneumonia.  **Foot conditions**  **Fungal nail or mycosis**  One CPG strongly recommended that treatment should be started to prevent ulcers and secondary bacterial infections for patients with a fungal nail or mycosis [46].  **Hyperkeratotic lesions**  One CPG strongly recommended that pressure and shearing forces should be normalised in feet with hyperkeratotic lesions. For normalisation of pressure and shearing forces:   - Advice on footwear and socks can be given [46]. - Foot orthoses, silicone toe orthosis, technical adaptations to over-the-counter shoes, ready- or custom-made therapeutic shoes, or a provisional therapy (e.g., felt padding or taping) can be prescribed [46].   **Excessive hyperkeratotic lesions**  One CPG strongly recommended that excessive hyperkeratotic lesions should be treated. During the treatment the following factors should be considered: i) a sensibility disorder or peripheral artery disease, and ii) fragile skin, plantar bursa, and prominent metatarsal heads on the foot of interest [46].  **Preulcer or infection**  One CPG strongly recommended that when a pre-ulcer or infection is detected, the treating physician should be consulted [46].  **Wound-care**  One CPG conditionally recommended that in wound-care, a provisional therapy (e.g., felt padding) can be applied to reduce pressure. When material with an adhesive layer is used, fragile skin should be taken into consideration [46].  **Pregnancy and lactation**  One CPG strongly recommended that biologic disease-modifying anti-rheumatic drug (bDMARDs) should only be considered after thorough assessment of benefits and risks for those who are pregnant or lactating [45].  **Cardiovascular disease (CVD)**  One CPG strongly recommended that all RA patients complete a CVD risk assessment at least once every 5 years and should be reconsidered following major changes in antirheumatic therapy. CVD risk estimation for patients with RA should be performed according to national guidelines and the SCORE CVD risk prediction model should be used if no national guideline is available. CVD risk prediction models should be adapted for patients with RA by a 1.5 multiplication factor, if this is not already included in the model [4].  One CPG conditionally recommended screening for asymptomatic atherosclerotic plaques by use of carotid ultrasound as part of the CVD risk evaluation in patients with RA [4].  Lifestyle recommendations should emphasize the benefits of a healthy diet, regular exercise and giving up smoking for all patients [4]. In CVD risk management, antihypertensives and statins may be used as in the general population. Prescription of non-steroidal anti-inflammatories should be given with caution, especially for patients with documented CVD or in the presence of CVD risk factors [4].  **Nontuberculous mycobacterial (NTM) lung disease**  One CPG conditionally recommended the following: [48]   - Use of the lowest possible dose of glucocorticoids (discontinuation if possible) over continuation of glucocorticoids without dose modification for patients with NTM lung disease. - The addition of conventional synthetic disease-modifying anti-rheumatic drug (csDMARDs) over addition of a bDMARD or targeted synthetic disease-modifying anti-rheumatic drug (tsDMARD) for patients who have moderate-to- high disease activity despite csDMARD monotherapy. - Abatacept over other bDMARDs and tsDMARDs for patients who have moderate-to- high disease activity despite csDMARDs   **Pulmonary disease**  One CPG conditionally recommended methotrexate (MTX) over alternative disease-modifying anti-rheumatic drugs (DMARDs) for the treatment of inflammatory arthritis for patients with clinically diagnosed mild and stable airway or parenchymal lung disease, or incidental disease detected on imaging, who have moderate-to- high disease activity [48].  **Non-alcoholic fatty liver disease (NAFLD)**  One CPG conditionally recommended MTX over alternative DMARDs for DMARD-naive patients with NAFLD, normal liver enzymes and liver function tests, and no evidence of advanced liver fibrosis who have moderate-to- high disease activity [48].  **Persistent hypogammaglobulinemia without infection**  One CPG conditionally recommended In the setting of persistent hypogammaglobulinemia without infection, continuation of rituximab therapy for patients at target over switching to a different bDMARD or tsDMARD [48].  **Subcutaneous nodules**  One CPG conditionally recommended MTX over alternative DMARDs for patients with subcutaneous nodules who have moderate-to-high disease activity [48]. Switching to a non-MTX DMARD is conditionally recommended over continuation of MTX for patients taking MTX with progressive subcutaneous nodules [48].  **Lymphoproliferative disorder**  One CPG conditionally recommended rituximab over other DMARDs for patients who have a previous lymphoproliferative disorder for which rituximab is an approved treatment and who have moderate-to- high disease activity [48]. |
| **Surgical** |
| **Education surgical benefits**  One CPG strongly recommended explaining the expected benefits of surgery for patients with RA, this includes: pain relief, improvement, or prevention of further deterioration, of joint function, and prevention of deformity [44].They highlight cosmetic improvements should not be the dominant concern [44].  **Medical and surgical management**  One CPG strongly recommended offering a combination of medical and surgical management urgently, to adults with RA who have suspected or proven septic arthritis (especially in a prosthetic joint) [44].  **MRI and surgical referral**  One CPG strongly recommended that if an adult with RA develops any symptoms or signs that suggest cervical myelopathy (for example, paraesthesia, weakness, unsteadiness, reduced power, extensor plantars), clinicians should request an urgent MRI scan, and refer for a specialist surgical opinion [44].  **Joint replacement surgery for younger adults**  One CPG conditionally recommended clinicians do not let concerns about the long-term durability of prosthetic joints influence decisions to offer joint replacements to younger adults with RA [44].  **Lower limb surgical interventions**  **Resection arthroplasty of the MTP joints**  One CPG conditionally recommended resection arthroplasty of the MTP joints to improve joint mobility and to reduce pain, forefoot plantar pressure, and problems finding well-fitting shoes. In severe malalignments of the toes or damage to the MTP joints, resection arthroplasty is preferred. Without severe malalignments/damage, a MTP joint-preserving surgical technique can be considered [46].  **Arthrodesis of the MTP1 joint**  One CPG conditionally recommended arthrodesis of the MTP1 joint to reduce pain and improve the weight-bearing capacity of the forefoot [46].  **Arthrodesis of the subtalar joint and/or arthrodesis of the calcaneocuboid joint and talonavicular joint**  One CPG strongly recommended arthrodesis of the subtalar joint when performing surgical treatment of the hindfoot is indicated. For flat feet, an additional arthrodesis of the calcaneocuboid joint and talonavicular joint should be considered (triple arthrodesis) [46].  **Arthrodesis of the tibiotalar joint or an ankle prosthesis**  One CPG conditionally recommended arthrodesis of the tibiotalar joint or an ankle prosthesis when the patient is experiencing severe pain and damage of the tibiotalar joint. An arthrodesis is preferred, provided that the Chopart-joint-line is intact and the status of other joints does not form a contraindication. An ankle prosthesis can be considered when preservation of mobility in the tibiotalar joint is important (according to the patient) and the preoperative status of the patient does not form a contra-indication [46]. |

bDMARD – biologic disease-modifying anti-rheumatic drug; CPG – clinical practice guideline; csDMARD – conventional synthetic disease-modifying anti-rheumatic drug; CVD – cardiovascular disease; DMARD – disease-modifying anti-rheumatic drug; MTX – methotrexate; MTP – metatarsophalangeal; NAFLD – non-alcoholic fatty liver disease; NTM – nontuberculous mycobacterial; RA – rheumatoid arthritis.
